# Adipose Tissue CIDEA Is Associated, Independently of Weight Variation, to Change in Insulin Resistance during a Longitudinal Weight Control Dietary Program in Obese Individuals

**DOI:** 10.1371/journal.pone.0098707

**Published:** 2014-07-01

**Authors:** Emilie Montastier, Sébastien Déjean, Caroline Le Gall, Wim H. M. Saris, Dominique Langin, Nathalie Viguerie

**Affiliations:** 1 Institut National de la Santé et de la Recherche Médicale (INSERM), UMR1048, Obesity Research Laboratory, Institute of Metabolic and Cardiovascular Diseases (I2MC), Toulouse, France; 2 University of Toulouse, UMR1048, Paul Sabatier University, Toulouse, France; 3 Toulouse University Hospitals, Departments of Clinical Biochemistry, Toulouse, France; 4 Toulouse University, Institut de Mathématiques UMR CNRS 5219, Toulouse, France; 5 Department of Human Biology, NUTRIM School for Nutrition, Toxicology and Metabolism, Maastricht University Medical Centre, Maastricht, the Netherlands; NIDDK/NIH, United States of America

## Abstract

**Aim:**

Weight loss reduces risk factors associated with obesity. However, long-term metabolic improvement remains a challenge. We investigated quantitative gene expression of subcutaneous adipose tissue in obese individuals and its relationship with low calorie diet and long term weight maintenance induced changes in insulin resistance.

**Research Design:**

Three hundred eleven overweight and obese individuals followed a dietary protocol consisting of an 8-week low calorie diet followed by a 6-month ad libitum weight-maintenance diet. Individuals were clustered according to insulin resistance trajectories assessed using homeostasis model assessment of insulin resistance (HOMA-IR) index. Adipose tissue mRNA levels of 267 genes selected for regulation according to obesity, metabolic status and response to dieting was assessed using high throughput RT-qPCR. A combination of discriminant analyses was used to identify genes with regulation according to insulin resistance trajectories. Partial correlation was used to control for change in body mass index.

**Results:**

Three different HOMA-IR profile groups were determined. HOMA-IR improved during low calorie diet in the 3 groups. At the end of the 6-month follow-up, groups A and B had reduced HOMA-IR by 50%. In group C, HOMA-IR had returned to baseline values. Genes were differentially expressed in the adipose tissue of individuals according to groups but a single gene, CIDEA, was common to all phases of the dietary intervention. Changes in adipose tissue CIDEA mRNA levels paralleled variations in insulin sensitivity independently of change in body mass index. Overall, CIDEA was up-regulated in adipose tissue of individuals with successful long term insulin resistance relapse and not in adipose tissue of unsuccessful individuals.

**Conclusion:**

The concomitant change in adipose tissue CIDEA mRNA levels and insulin sensitivity suggests a beneficial role of adipose tissue CIDEA in long term glucose homeostasis, independently of weight variation.

**Trial Registration:**

ClinicalTrials.gov NCT00390637

## Introduction

Insulin resistance (IR) and type 2 diabetes are strongly associated with excess fat mass. This is reversible with weight loss [1]. However, the extent to which weight loss reduces IR is heterogeneous and the improvement in IR is not stable over time [2].

The adipose tissue (AT) is a tissue devoted to energy storage as triglycerides. An overload of the buffering capacities of AT leads to a pro-inflammatory, diabetogenic and atherogenic status [3]. Thus, AT represents a key tissue in the study of obesity-related complications.

Carefully monitored weight-control diets favorably affect parameters of the metabolic syndrome and delay the onset of diabetic complications. As a main target tissue during dietary interventions, adaptations occurring in AT are likely to have a profound impact on the whole body response.

Up to now, gene expression remains the most powerful method to comprehensively explore molecular adaptations from minute amounts of tissue [4]. Pattern of gene expression has shown promising potential for identifying undiscovered aspects of the secretory and metabolic aspects of various tissues including AT [5].

Here, we investigated whether individuals respond differently to the same dietary intervention regarding IR. We hypothesized that AT gene profiling would help identifying novel biomarkers that underline long term changes in IR with weight loss.

## Methods

### Ethics Statement

The project Diet, Obesity and Genes (DiOGenes) is a pan-European study which was approved by the ethics committees of each of the 8 European centers participating to the program. Written informed consent was obtained from each patient according to the local ethics committee of the participating countries: 1, Medical Ethics Committee of the University Hospital Maastricht and Maastricht University, The Netherlands; 2, The Committees on Biomedical Research Ethics for the Capital region of Denmark, Denmark; 3, Suffolk Local Research Ethics Committee, UK; 4, University of Crete Ethics Committee, Greece; 5, the Ethics Commission of the University of Potsdam; 6, Research Ethics Committee at the University of Navarra, Spain; 7, Ethical Committee of the Institute of Endocrinology, Czech Republic; 8, Ethical Committee to the National Transport Multiprofile Hospital in Sofia, Bulgaria.

### Intervention trial

**Figure 1:**
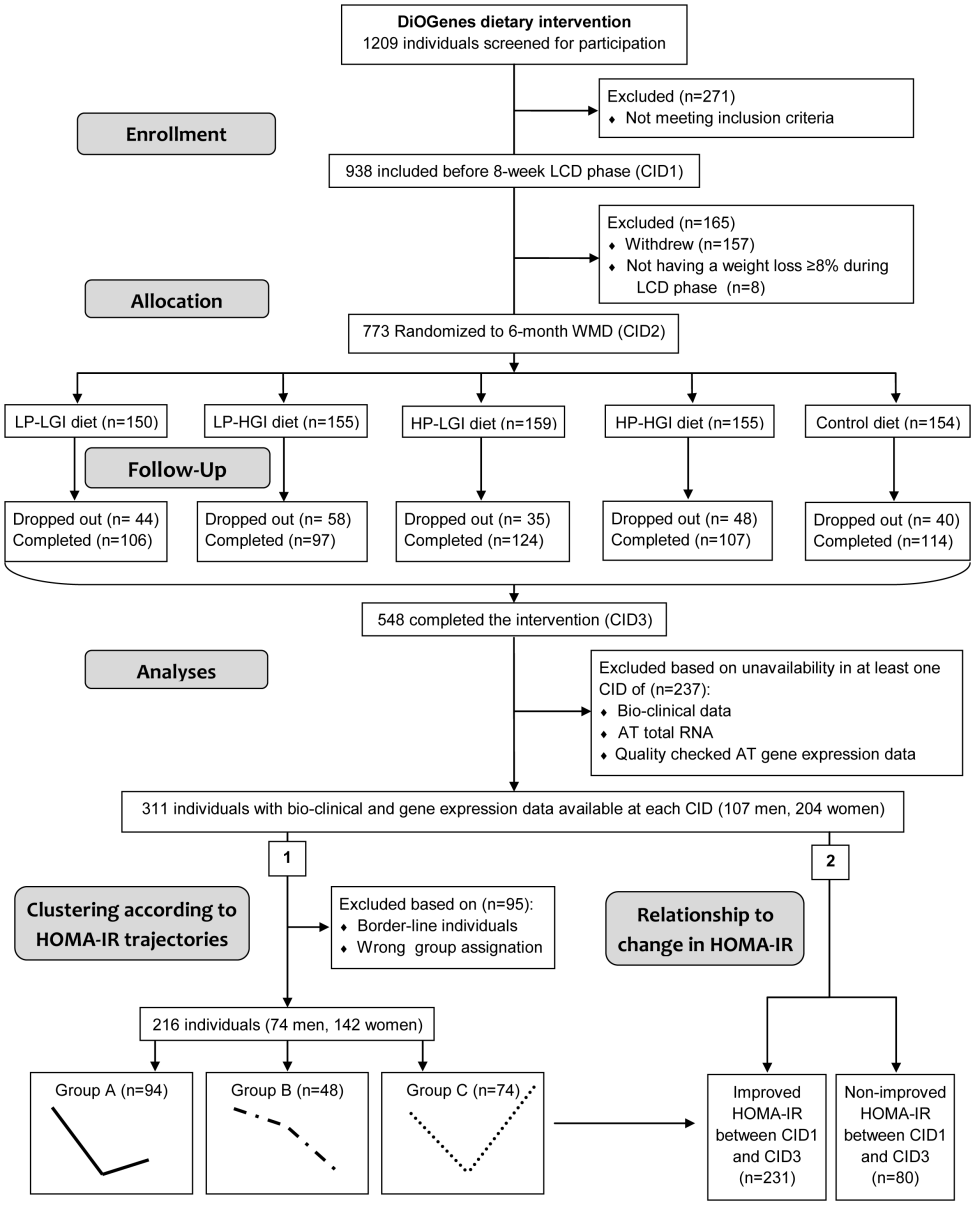
Flowchart for individuals' selection from the DiOGenes cohort. Participants entering subsequent phases of the study as well as dropouts are indicated in total. AT, adipose tissue; CID, clinical investigation day; HGI, high glycemic index; HOMA-IR, Individual Homeostasis Model Assessment of Insulin Resistance; HP, high protein; LCD, low calorie diet; LGI, low glycemic index; LP, low protein; WMD, weight maintenance diet.

DiOGenes is a multicentre dietary intervention study. The protocol for this trial and supporting CONSORT checklist are available as supporting information; see Checklist S1 and Protocol S1. The DiOGenes study started January 2005 and enrollment began November 2005 in the first two centres, followed later in the autumn of 2006 of the other 6 centres. In 2006, clinical trials registration became highly recommended and ClinicalTrials.gov registry DiOGenes certification occurred October 2006 (registration no. NCT00390637), before registration became mandatory according to the 59th World Medical Association general assembly (Seoul, Republic of Korea, October 2008). Design and dietary intervention have been described in detail previously [6] (Protocol S1 and Checklist S1). Briefly, from January 2006 to August 2007, overweight and obese adults participated in a dietary program with 8-week low calorie (3.3–4.2 MJ/d) diet (LCD) using commercial meal replacements (Modifast, Nutrition et Santé, France) and up to 400 g of vegetables. Individuals achieving at least 8% of initial body weight loss were randomized to a 6-month weight maintenance with *ad libitum* diet (WMD) consisting in one of four reduced fat diets that differed in glycemic index of carbohydrates (low or high) and protein content (low or high), or a control diet according to National Dietary Guidelines in the participating countries. Target intakes in the low protein diets were 10–15% energy intake for proteins and 57–62% for carbohydrates and, in high protein diets, 23–28% energy intake for proteins and 45–50% energy intake for carbohydrates. During WMD, in 2 centers, the individuals were provided dietary instruction plus free foods. At the remaining 6 centers, they received dietary instruction. Dietary intake was assessed at screening (prior to baseline), four weeks after the beginning of WMD and at the end of WMD. The subjects were asked to complete a 3-day weighed food record, including 2 week days and 1 week-end day. All dietary records were validated by a nutritionist. Despite low glycemic index diets were targeted to a reduction of 15 glycemic index points compared with the high glycemic index diets, the mean glycemic index in the low glycemic index groups was only 5 units lower than that in the high glycemic index groups [6]. Physical activity was assessed using Baecke questionnaire that discriminate 3 distinct dimensions: work, sports and leisure activities [7]. Physical activity was calculated as the sum of the individual Baecke indexes. At each clinical investigation day (CID) (i.e. at baseline (CID1), after LCD (CID2) and after WMD (CID3)), bio-clinical data were collected, blood was sampled and a needle biopsy of abdominal subcutaneous AT was performed. Detailed method for sampling and data collection in the DiOGenes trial has previously been published [8]. IR was assessed using the homeostatic model assessment (HOMA-IR) calculated as [fasting glucose (mM)×fasting insulin (mU/l)/22.5] [9]. The authors confirm that all ongoing and related trials for this intervention are registered. Figure 1 displays the organizational flowchart through the trial protocol and the individuals' selection from the DiOGenes cohort for the present study.

### Adipose tissue gene expression

The 267 genes, the corresponding biological functions and the strategy used for selection are summarized in Table S1. These transcripts were selected from previous published and unpublished DNA microarray analyses on limited number of individuals as described in [10]. The list of genes extracted from microarray data includes 102 genes previously shown as markers of subcutaneous AT from obese insulin resistant subjects with metabolic syndrome [11], 56 genes described as markers of subcutaneous AT from lean individuals [11], 44 markers of weight changes after caloric restriction [12], 40 genes selected from previous caloric restriction induced weight loss studies [13], [14], and 25 unpublished predictors of weight change to distinguish between those subjects that will regain weight after LCD from those that will succeed weight maintaining based on the AT transcriptome at baseline or after the caloric restriction phase. These genes encoded proteins involved in various pathways including metabolism (40% of the transcripts), immune response (22%), signal transduction (17%), cell and tissue structure (5%), transport (4%) and response to stress (2%).

Total RNA isolation, cDNA synthesis and massive parallel real-time PCR using microfluidic qPCR device (Biomark Dynamic Array, Fluidigm) were performed as previously described [10]. Messenger RNA levels were normalized to *GUSB* mRNA, using the comparative cycle threshold (Ct), 2^−ΔΔCt^ method. The present study was based on a subgroup of 311 obese individuals (107 men and 204 women) with bio-clinical and adipose tissue gene expression data available at each CID (Figure 1).

### Statistical analysis

R software (version 2.14.0) was used for hierarchical clustering, Random Forests (RF), sparse Partial Least Square Discriminant Analysis (sPLS-DA) and partial correlations, along with different R packages. Conventional statistical analyses (Kolmogorov–Smirnov, ANOVA, Kruskal-Wallis, Wilcoxon, Mann and Whitney and χ^2^ tests) were carried out with SPSS Statistics version 17.0 for windows (SPSS Inc., Chicago, Ill).

Individuals' grouping was performed using hierarchical clustering analysis on a matrix composed of two synthetic variables, V1 and V2. V1 was calculated as the difference in HOMA-IR between end of LCD and baseline, and V2 as the difference in HOMA-IR between the end of WMD and the end of LCD. The Euclidean distance was used as a measure of similarity and the Ward's method was chosen as agglomeration criteria. The dendrogram obtained was cut in 3 groups. Groups were then hand-cleaned from border line individuals and wrong group assignation (based on HOMA-IR trajectories) to get more homogeneous clusters resulting in a final analysis of 216 individuals.

Gaussian distribution of data was tested using the Kolmogorov–Smirnov test. Group comparisons were made using the χ^2^, one-way ANOVA (normally distributed data), Kruskal-Wallis, Wilcoxon (paired data) and Mann and Whitney (unpaired data) tests (non-normal data) with Bonferroni correction. Significance cut-off was set at 0.01 which is lower than the Bonferroni adjusted p value (0.0167).

Two multivariate discriminant analyses were used to identify the important variables (i.e. mRNA levels) for group classification: RF (R package : *randomForest*) and sPLS-DA (R package : *mixOmics*). RF is an ensemble of classification trees calculated on random subsets of the data, using a subset of randomly selected variables for each split in each classification tree. Mean Decreased Gini (MDG) is used as an indicator of importance of the variables. The sPLS-DA is based on the PLS approach in which there is only one dependent variable chosen to represent the class/group membership. The variable influence on projection (VIP) indicates the influence of each variable on the discrimination between the different groups. These two methods were chosen because of their complementarity: sPLS-DA is a method taking place in a linear framework whereas RF is a nonlinear one. To improve classification power, when comparing baseline to the end of the dietary intervention, groups A and B (individuals with reduction in IR) were compared to group C. During LCD and WMD, because the variations in HOMA-IR were different in the 3 groups, no group aggregation was made. For each phase of the dietary intervention, the most important transcripts in the classification were ranked according to decreasing VIP or MDG. A classification score was calculated as the sum of the rank for each gene. Partial correlations were computed to assess direct correlations independently of other confounding variable, i.e. change in body mass index (BMI) [15].

## Results

### Clustering of obese subjects according to insulin resistance trajectories

**Figure 2:**
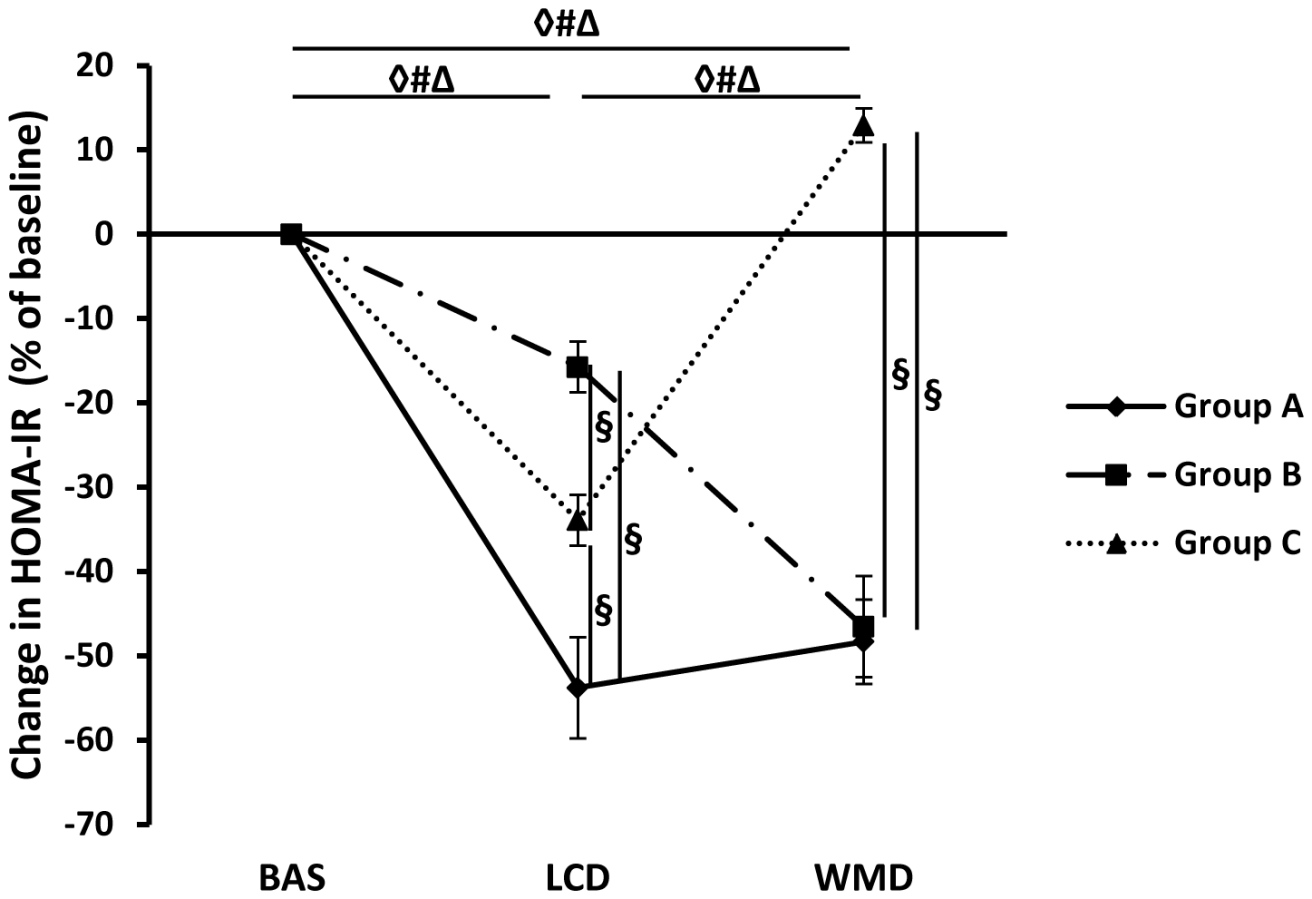
Clustering of insulin resistance profiles in obese individuals during the dietary intervention (n = 216). Individual Homeostasis Model Assessment of Insulin Resistance (HOMA-IR) changes during an 8-week calorie restriction (LCD) and a 6-month weight maintenance diet (WMD) were clustered, resulting in 3 groups. The panel shows mean HOMA-IR changes expressed as change after LCD and at the end of the 6-montho weight maintenance (WMD) *vs.* baseline (BAS) values according to groups. The black line shows the HOMA-IR profile for group A (n = 94). The dashed line shows the HOMA-IR profile for group B (n = 48). The dotted line shows the HOMA-IR profile for group C (n = 74). The A, B, and C are the mean of HOMA-IR changes in each clustered group. **◊**: p<0.05, data different in group A. Δ: p<0.05, data different in group B. #: p<0.05, data different in group C. §: p<0.05, data difference between groups. Values are means ± SEM.

Clustering analysis identified 3 groups of individuals (total number  = 216) which showed different profiles of change in HOMA-IR during the whole dietary intervention (Figure 2).

At baseline, there was no significant between group difference in bio-clinical parameters except for blood pressure, fasting plasma glucose and insulin, and HOMA-IR (Table 1). Groups A, B and C encompassed 62, 42 and 35% of IR individuals, respectively, with a value >2.3 defining IR state in obese individuals [16]. Proportions of IR individuals were different between groups A and C. Individuals from group C had higher blood pressure than those from group A or B, but overall were normotensive. No intergroup difference was found for center, sex, or diet during WMD (data not shown).

**Table 1:** Bioclinical parameters of the 3 groups at baseline.

	Group A	Group B	Group C	P-value
n	94	48	74	
men/women	34/60	12/36	28/46	
Age (years)	43±6	42±7	42±6	0.81
Total Energy Intake (kJ/d)	8734±3027	8780±2682	9787±3728	0.08
Physical Activity	8.2±0.8	8.1±0.9	7.9±0.9	0.29
HOMA-IR	3.3±2.0	2.4±1.3	2.3±1.1	a ^, ^ b ^, ^ c
Glucose (mmol/l)	5.3±0.7	5.0±0.7	5.1±0.6	a ^, ^ b ^, ^ c
Insulin (µIU/ml)	13.7±7.0	10.7±5.4	10.0±4.6	a ^, ^ b ^, ^ c
Weight (kg)	99.6±19.6	99.2±14.8	101.5±17.0	0.43
BMI (kg/m^2^)	34.4±4.8	34.8±4.7	35.0±5.2	0.62
Waist (cm)	108.7±13.7	105.3±12.3	108.1±11.6	0.36
Fat Mass (%)	40.0±7.5	42.3±8.3	39.2±8.7	0.33
SBP (mmHg)	126.7±14.0	120.9±14.2	131.0±15.7	a ^, ^ d
DBP (mmHg)	76.5±10.5	73.1±11.9	80.8±11.4	a ^, ^ c ^, ^ d
Triglycerides (mmol/l)	1.4±0.6	1.2±0.6	1.5±0.7	0.13
Total Cholesterol (mmol/l)	5.0±1.1	4.7±1.0	4.8±0.9	0.65
HDL-C (mmol/l)	1.2±0.3	1.2±0.3	1.2±0.3	0.32
LDL-C (mmol/l)	3.1±0.9	2.9±0.9	2.9±0.8	0.56
Adiponectin (µg/ml)	8.9±3.6	9.7±3.3	9.5±5.3	0.22
CRP (mg/ml)	4.2±3.8	3.4±2.5	4.0±3.7	0.73
Fructosamine (µM)	206.0±25.5	206.9±31.0	209.4±19.3	0.73

χ^2^ test was used for comparison between observed and expected distribution. Kruskal-Wallis rank sum test stands for variance among 3 clusters:

a , significant difference among the 3 groups. Significance is set at 0.05. Mann and Whitney test stands for comparison between 2 groups:

b , significant difference between A and B;

c , significant difference between A and C.

d , significant difference between B and C. Significance is set at 0.01, after adjustment for multiple comparisons.

HOMA-IR, Homeostasis Model Assessment of Insulin Resistance; SBP, systolic blood pressure; DBP, diastolic blood pressure; CRP, C reactive protein. Values are means ± SD.

Changes in bio-clinical characteristics during the 2 phases of the dietary intervention according to groups are displayed in Table 2. During LCD, HOMA-IR significantly decreased in all groups. During WMD, HOMA-IR further decreased in group B and increased in groups A and C. At the end of dietary intervention, groups A and B had a lower HOMA-IR than at baseline, and group C had slightly higher HOMA-IR than baseline (Figure 2). During WMD total energy intake slightly increased. There was no group difference in changes in dietary intake during the two phases. There was a slight increase in physical activity during LCD in groups A and C, but no significant change during WMD. During LCD, anthropometric parameters decreased similarly in the 3 groups and remained stable during WMD. There was no intergroup difference in weight change during the entire dietary intervention. No intergroup difference was found for total dietary intake or physical activity at each clinical investigation day (data not shown).

**Table 2:** Fold changes in bioclinical parameters according to the 3 groups during low calorie diet and during weight maintenance diet.

	LCD				WMD			
	Group A	Group B	Group C	P-value	Group A	Group B	Group C	P-value
Total Energy Intake (kJ/d)	0.74±0.33^a^	0.67±0.27^a^	0.74±0.37^a^	0.50	1.18±0.43^a^	1.27±0.35^a^	1.31±0.54^a^	0.11
Physical Activity	1.03^a^±0.09	1.02±0.08	1.04^a^±0.09	0.83	1.03±0.10	1.02±0.08	0.99±0.07	0.41
HOMA-IR	0.50±0.18^a^	0.90±0.23^a^	0.69±0.26^a^	b ^,^ c ^,^ d ^,^ e	1.17±0.35^a^	0.63±0.14^a^	1.88±0.79^a^	b ^,^ c ^,^ d ^,^ e
Glucose (mmol/l)	0.90±0.08^a^	1.00±0.12^a^	0.96±0.09^a^	b ^,^ c ^,^ d	1.04±0.09^a^	0.97±0.08^a^	1.07±0.10^a^	b ^,^ c ^,^ e
Insulin (µIU/ml)	0.55±0.20^a^	0.90±0.23^a^	0.72±0.26^a^	b ^,^ c ^,^ d ^,^ e	1.12±0.31^a^	0.65±0.14^a^	1.75±0.67^a^	b ^,^ c ^,^ d ^,^ e
Weight (kg)	0.89±0.03^a^	0.89±0.02^a^	0.89±0.02^a^	0.37	1.00±0.05	1.00±0.07	1.01±0.06^a^	0.07
BMI (kg/m^2^)	0.89±0.03^a^	0.89±0.02^a^	0.89±0.02^a^	0.37	1.00±0.05	1.00±0.07	1.01±0.06^a^	0.06
Waist (cm)	0.91±0.04^a^	0.92±0.04	0.91±0.04^a^	0.54	1.01±0.06	1.01±0.07	1.01±0.07	0.62
Fat Mass (%)	0.88±0.01^a^	0.89±0.02^a^	0.91±0.01^a^	0.07	0.99±0.09	0.97±0.08^a^	0.99±0.12	0.43
SBP (mmHg)	0.93±0.08^a^	0.95±0.07^a^	0.94±0.08^a^	0.43	1.04±0.10^a^	1.05±0.11^a^	1.03±0.11^a^	0.92
DBP (mmHg)	0.93±0.01^a^	0.95±0.01^a^	0.94±0.01^a^	0.65	1.04±0.12^a^	1.03±0.10	1.03±0.12	0.97
Triglycerides (mmol/l)	0.78±0.31^a^	0.93±0.39^a^	0.92±0.38^a^	b	1.32±0.53^a^	1.10±0.41	1.14±0.41	b ^,^ c
Total Cholesterol (mmol/l)	0.85±0.01^a^	0.90±0.03^a^	0.89±0.01^a^	b	1.25±0.29^a^	1.16±0.27^a^	1.20±0.21^a^	0.11
HDL-Cholesterol (mmol/l)	1.02±0.02	0.95±0.03	0.95±0.02^a^	b ^,^ c	1.21±0.23^a^	1.23±0.23^a^	1.19±0.19^a^	0.66
LDL- Cholesterol (mmol/l)	0.83±0.02^a^	0.90±0.03	0.89±0.02^a^	b	1.28±0.41^a^	1.16±0.35^a^	1.22±0.32^a^	0.14
Adiponectin (µg/ml)	1.24±1.33^a^	1.24±1.56^a^	1.23±1.44	0.17	1.16±0.39^a^	1.22±0.41^a^	1.21±0.33^a^	0.35
CRP (mg/ml)	1.09±2.83^a^	0.96±0.99^a^	0.90±0.67^a^	0.67	1.12±0.87^a^	1.97±3.82	1.30±1.50	0.94
Fructosamine (µM)	1.02±0.01	1.04±0.03^a^	1.01±0.01	0.96	1.06±0.12^a^	1.03±0.14	1.02±0.08	0.17

Fold changes were calculated as ratio between the end of LCD and baseline (LCD phase), and the end of WMD and the end of LCD (WMD phase). Wilcoxon test stands for comparison between each time point:

a , significant between baseline and the end of LCD or between the beginning and the end of WMD. Kruskal-Wallis rank sum test stands for variance among 3 groups during each phase:

b , significant difference among 3 groups. Significance is set at 0.05. Mann and Whitney test stands for comparison between 2 groups during each phase (LCD, low calorie diet or WMD, weight maintenance diet):

c , significant difference between A and B;

d , significant difference between A and C;

e , significant difference between B and C. Significance is set at 0.01, after adjustment for multiple comparisons.

HOMA-IR, Homeostasis Model Assessment of Insulin Resistance; LCD, low calorie diet; SBP, systolic blood pressure; DBP, diastolic blood pressure; CRP, C reactive protein; WMD, weight maintenance diet. Values are means ± SD.

### Variation in HOMA-IR during dietary intervention is related to adipose tissue CIDEA

The relationship between AT gene expression and the HOMA-IR response in the 3 groups was investigated using sPLS-DA and RF during each phase of the dietary intervention. Genes with the top 5 classification scores were considered (Table 3). *CIDEA* was found in top 5 ranking in all phases. In order to control for body weight changes, partial correlations were computed. Only genes with significant class prediction potency independent of changes in BMI were taken into account. When adjusting for change in BMI, *CIDEA* was the only gene with change in mRNA levels allowing distinction of individuals with alleviated IR from those who did not.

**Table 3:** List of top 5 genes for classification.

DI	Score	LCD	Score	WMD	Score
PCK2	4	NPAS3	6	PKM2	3
E2F4	5	SLC19A2	12	**CIDEA**	16
ACTR3	12	C1QB	21	EMILIN2	16
**CIDEA**	13	GATM	24	ENO1	17
LDLR	17	**CIDEA**	25	GAPDH	22

DI, dietary intervention; LCD, low calorie diet; WMD, weight maintenance diet.

Score is the mean of the ranking from sparse Partial Least Square Discriminant Analysis and Random Forests based on Variable Influence on Projection and Mean Decreased Gini, respectively. Bold indicate genes with correlation independent from change in body mass index (n = 216).

**Figure 3:**
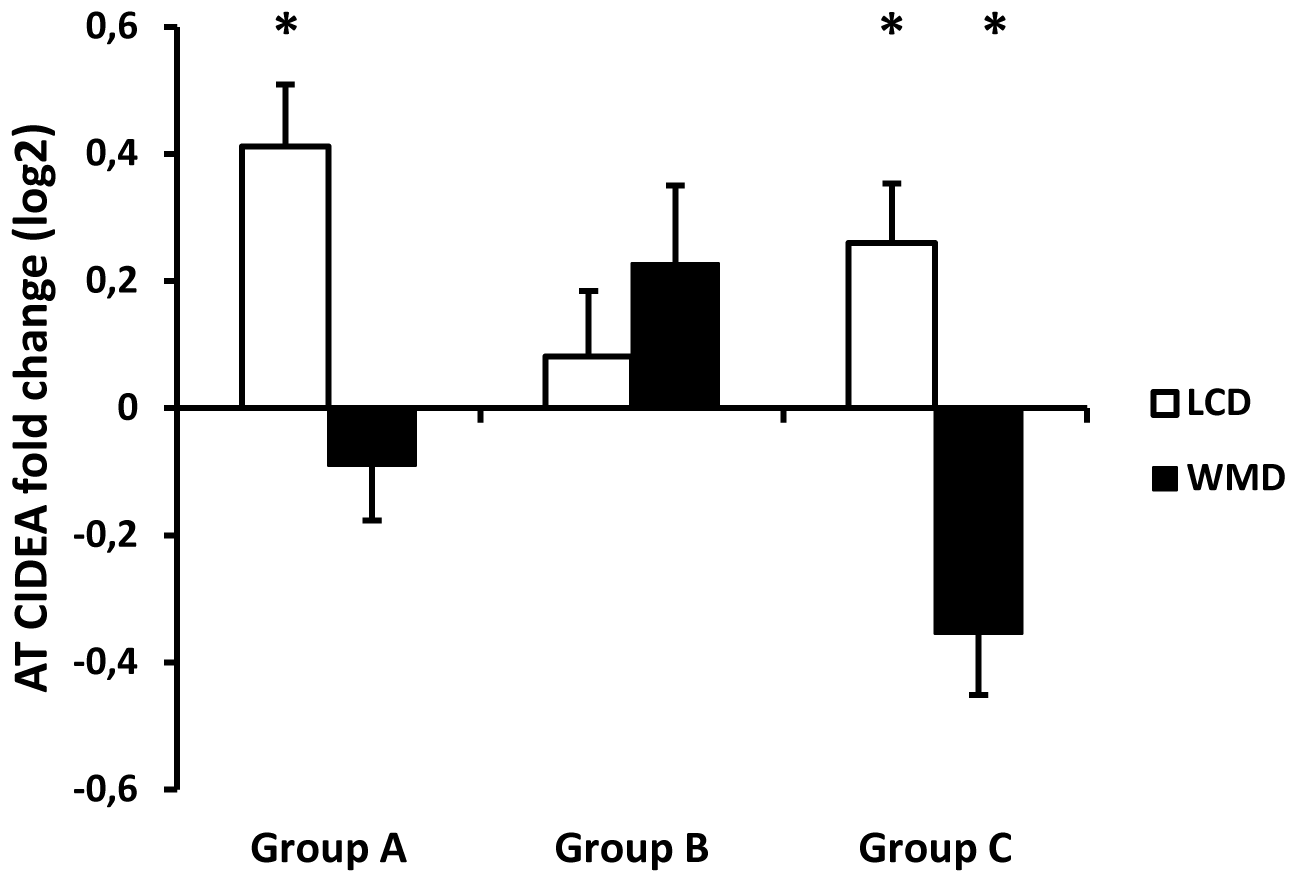
Human adipose tissue CIDEA gene expression according to insulin resistance profiles (n = 216). Relative *CIDEA* mRNA levels in subcutaneous adipose tissue were plotted according to groups compared to baseline, after 8 weeks low calorie diet (LCD, white bars), and after 6 months of weight maintenance diet (WMD, black bars). AT, adipose tissue; HOMA-IR, Homeostasis Model Assessment of Insulin Resistance. *: p<0.05, difference between baseline and the end of LCD or WMD. Values are means ± SEM.

The changes in *CIDEA* mRNA levels according to groups during dietary intervention mirrored the evolution of insulin sensitivity (Figure 3). During LCD, AT *CIDEA* mRNA levels increased in groups A and C. During WMD, *CIDEA* mRNA levels decreased in group C only. Overall, from baseline to WMD, *CIDEA* mRNA levels increased only in group A.

**Figure 4:**
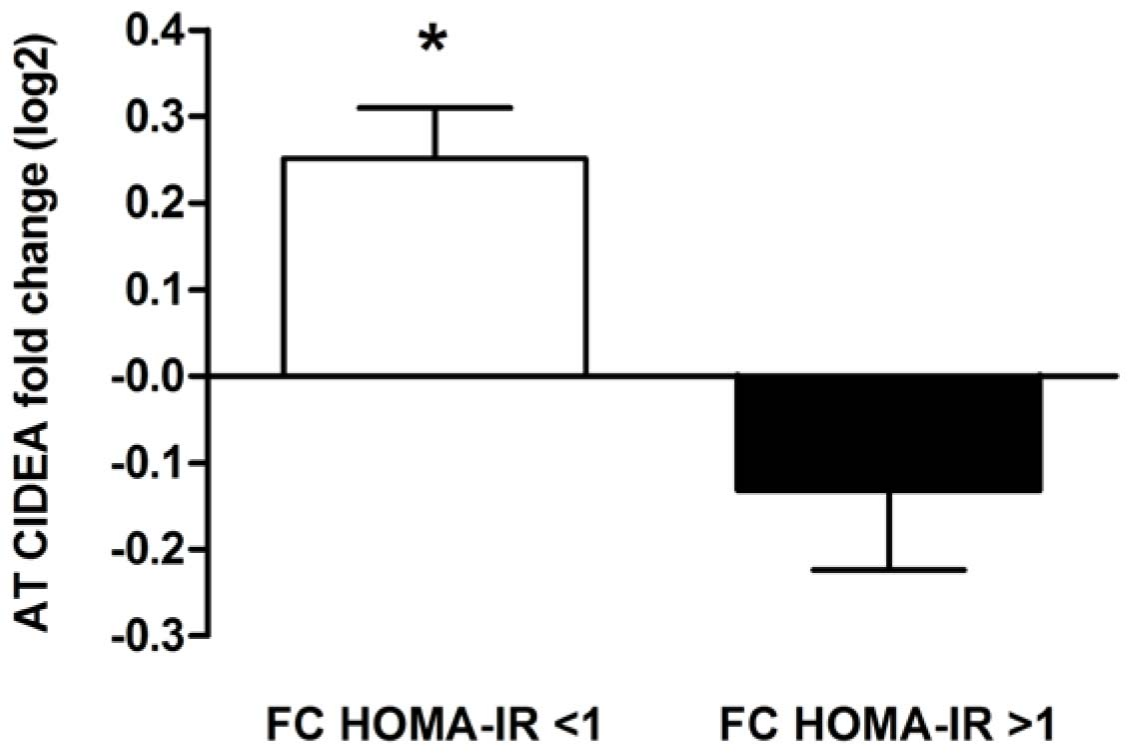
Adipose tissue CIDEA gene expression according to improvement of insulin sensitivity during dietary intervention (n = 311). CIDEA mRNA levels fold change (FC) in subcutaneous adipose tissue were plotted according to FC in HOMA-IR during the entire dietary intervention. AT, adipose tissue; HOMA-IR, Homeostasis Model Assessment of Insulin Resistance. *: p<0.05, difference between baseline and the end of dietary intervention. Values are means ± SEM.

In the whole data set of 311 individuals, we could explore the relationship between changes in AT *CIDEA* mRNA levels and variations in HOMA-IR during the entire dietary intervention. Two hundred thirty one individuals improved insulin sensitivity (fold change HOMA-IR <1) and 80 had no decrease in HOMA-IR between baseline and the end of WMD (fold change HOMA-IR >1). As shown on figure 4, individuals that improved insulin sensitivity during dietary intervention increased AT *CIDEA* mRNA levels by 47% (p value<0.0001) while those who returned to the original HOMA-IR value had no significant change in *CIDEA* gene expression (p value = 0.228).

## Discussion

Hypocaloric weight-reducing diets improve the metabolic profile of obese patients. However, the reasons for the limited longstanding success of dieting remain poorly understood. Here we focused on AT gene profiling and show regulation of *CIDEA* as associated to changes in IR independently of weight change during a two-phase longitudinal dietary program including a low calorie diet-induced weight reduction and subsequent follow-up.

The present analysis is an ancillary study to the DiOGenes trial which focused on body weight control after caloric restriction [6]. Here, we studied a subgroup of the DiOGenes cohort and focused on changes in IR. The IR was assessed using the HOMA-IR index which is calculated from fasting plasma glucose and insulin. HOMA-IR primarily reflects hepatic insulin resistance, while the hyperinsulinemic euglycemic glucose clamp mainly reflects muscle insulin resistance [17]. Despite HOMA-IR is not the gold standard method to assess insulin sensitivity compared to the hyperinsulinemic euglycemic glucose clamp it is an easy-to-use tool which has proved robust in large-scale clinical trials [18], [19].

First, 3 groups were defined according to HOMA-IR profiles during the two phases of the dietary intervention, resulting in the study of 216 individuals sourced from the 311 obese patients group (Figure 1). There was no difference in daily calorie intake between the 3 groups at baseline, after LCD and after WMD. Metabolic status of volunteers at baseline was similar in each group, except systolic and diastolic blood pressures that were higher in group C. This could argue for a higher susceptibility to type 2 diabetes in individuals from group C. However, these ranges of systolic and diastolic blood pressure are considered as normal, since they remain smaller than 140/90 mmHg [20].

Then, the use of discriminant analyses to find the transcripts most related to subject's grouping showed *CIDEA* as the most consistent transcript related to groups. The changes in *CIDEA* mRNA levels mirrored changes in insulin sensitivity. To further extend and validate the relationship of AT *CIDEA* expression to insulin sensitivity the whole data set of 311 individuals was investigated according to change in HOMA-IR among the entire dietary intervention (from baseline to the end of WMD), independently of groups.

*CIDEA* encodes a member of the cell death–inducing DNA fragmentation factor 45-like effector (CIDE) proteins, which is expressed in both brown and white fat [21]. Originally described as involved in apoptosis, CIDEA plays important roles in the development of metabolic disorders [22] and lipid metabolism [23].

A higher *CIDEA* mRNA level was found in the AT from obese individuals compared to lean ones [24]. Transcriptome studies of human AT also showed *CIDEA* as the most up-regulated gene after calorie restriction induced weight loss [25] that returned to baseline during weight maintenance [26] and was down-regulated with overfeeding [27]. The calorie restriction induced up-regulation was specific to individuals successful in body weight maintenance compared to those regaining the weight they had lost [28]. The reversal of *CIDEA* expression after dieting when calories are reintroduced while body weight is stable, shown in a subgroup of 40 obese women, indicates that during diet-induced weight loss, calorie restriction *per se*, rather than weight reduction, has the major impact on *CIDEA* expression [29].

Here we found a negative relationship between regulation of *CIDEA* in AT and change in IR both during (LCD) and after calorie restriction (WMD). The link between AT *CIDEA* and insulin sensitivity in obese humans has been described in a cross-sectional study showing higher *CIDEA* mRNA levels in AT of insulin-sensitive compared to BMI-matched IR obese individuals [16]. The present study is based on a two-phase longitudinal trial including calorie restriction and long-term follow-up. The strength of this study is that the AT gene profiling is based on a large cohort of carefully characterized individuals. Another mainstay is the study design of the trial that includes an *ad libitum* follow-up corresponding to a program with clinical relevance to obese individuals.

At the end of the calorie restriction phase, groups with largest IR improvement of had higher *CIDEA* mRNA level in AT, compared to baseline. During the second phase 6-month follow-up, only the group who increased HOMA-IR again showed a decreased *CIDEA* gene expression. The independence of the relationship between change in insulin sensitivity and AT *CIDEA* regulation from body mass change is noteworthy.

The observation that CIDEA is an AT marker of insulin sensitivity raises the question of the potential role of *CIDEA* in insulin sensitivity but does not establish a cause and effect relationship. However, previous investigations provide some evidence on the functional effect of *CIDEA* regulation in AT.

CIDEA is a multifunctional protein probably depending on intracellular localization. Overexpression in 3T3-L1 cells induced an increase in fatty acid oxidation and decrease in glucose transport [30]. CIDEA co-localizes with perilipins, which are lipid droplet proteins [16]. Fat droplet proteins regulate many cellular but also whole body processes [31], [32]. *Cidea* null mice have smaller adipocytes and are resistant to diet-induced obesity [33]. In human white preadipocytes, depletion of *CIDEA* increased basal lipolysis, supporting the idea that CIDEA protects lipid droplet [24]. Overexpression promoted triglyceride accumulation [34]. In a cohort of 367 individuals, the AT lipolytic capacity was positively associated to HOMA-IR [35]. When adjusted for BMI, a significant part of the variance in HOMA-IR remained explained by AT lipolysis. Furthermore, in a 2-year follow-up of obese patients after bariatric surgery, the higher was the decrease in AT lipolytic capacity the stronger was the improvement in IR [35]. In the present study, *CIDEA* regulation during dietary intervention was independent of body mass. Altogether, the increase in AT *CIDEA* mRNA levels is likely to explain IR improvement through a decreased AT lipolysis or fatty acids oxidation. The AT lipolytic capacities of the individuals enrolled in the present dietary program has not been studied during the course of the present trial. The investigation of a potential link between IR and AT CIDEA deserve future research.

One can hypothesize that change in IR observed during dietary intervention is due to CIDEA overexpression-induced increase in fatty acid oxidation and/or decreased lipolytic capacities. The present study cannot answer the question whether *CIDEA* regulation is causative or consequence of changes in IR. Nevertheless, this is the first report of a link between human AT *CIDEA* regulation and insulin sensitivity, independently of weight change. Collectively, these results provide new data that *CIDEA* regulation in AT is related to insulin sensitivity. The potential of CIDEA induction in the treatment of obesity-related complications deserve future research.

## Supporting Information

Table S1 **Description of the 267 target genes.**
(DOCX)

Protocol S1 **Detailed study protocol of the DiOGenes trial.**
(PDF)

Checklist S1 **CONSORT checklist of the DiOGenes trial.**
(DOC)
