# Supplementary material for: Adipose Tissue CIDEA Is Associated, Independently of Weight Variation, to Change in Insulin Resistance during a Longitudinal Weight Control Dietary Program in Obese Individuals
Source: PLoS One. 2014 Jul 1;9(7):e98707. doi: 10.1371/journal.pone.0098707 (PMC4077708; doi:10.1371/journal.pone.0098707)
Supplement: Table S1 — Description of the 267 target genes. (DOCX) [file pone.0098707.s001.docx]

| Gene Symbol | Gene Name | Biological Function |
| --- | --- | --- |
| **Amino Acid Metabolism** | | |
| *ALDH6A1* | aldehyde dehydrogenase 6 family, member A1 | amino acid catabolism |
| *BCAT1* | branched chain aminotransferase 1, cytosolic | amino acid catabolism |
| *BCKDHB* | branched chain keto acid dehydrogenase E1, beta polypeptide | amino acid catabolism |
| *CTH* | cystathionase (cystathionine gamma-lyase) | amino acid synthesis |
| *GPT* | glutamic-pyruvate transaminase (alanine aminotransferase) | amino acid synthesis |
| *GPT2* | glutamic pyruvate transaminase (alanine aminotransferase) 2 | amino acid synthesis |
| *KYNU* | kynureninase (L-kynurenine hydrolase) | amino acid catabolism |
| **Carbohydrate Metabolism** | | |
| *ADH1A* | alcohol dehydrogenase 1A (class I), alpha polypeptide | aerobic glycolysis |
| *ADH1C* | alcohol dehydrogenase 1C (class I), gamma polypeptide | aerobic glycolysis |
| *ALDOB* | aldolase B, fructose-bisphosphate | aerobic glycolysis |
| *ALDOC* | aldolase C, fructose-bisphosphate | aerobic glycolysis |
| *ENO1* | enolase 1, (alpha) | aerobic glycolysis |
| *ENO3* | enolase 3 (beta, muscle) | aerobic glycolysis |
| *FBP1* | fructose-1,6-bisphosphatase 1 | aerobic glycolysis |
| *GAPDH* | glyceraldehyde-3-phosphate dehydrogenase | aerobic glycolysis |
| *GBA* | glucosidase, beta, acid | glycolipid metabolism |
| *GYS1* | glycogen synthase 1 (muscle) | glycogenesis |
| *HK1* | hexokinase 1 | aerobic glycolysis |
| *LDHA* | lactate dehydrogenase A | anaerobic glycolysis |
| *PC* | pyruvate carboxylase | gluconeogenesis |
| *PCK1* | phosphoenolpyruvate carboxykinase 1 (soluble) | aerobic glycolysis |
| *PCK2* | phosphoenolpyruvate carboxykinase 2 (mitochondrial) | aerobic glycolysis |
| *PDHA1* | pyruvate dehydrogenase (lipoamide) alpha 1 | aerobic glycolysis |
| *PFKFB1* | 6-phosphofructo-2-kinase/fructose-2,6-biphosphatase 1 | aerobic glycolysis |
| *PFKM* | phosphofructokinase, muscle | aerobic glycolysis |
| *PGAM1* | phosphoglycerate mutase 1 (brain) | aerobic glycolysis |
| *PGK1* | phosphoglycerate kinase 1 | aerobic glycolysis |
| *PGM1* | phosphoglucomutase 1 | aerobic glycolysis |
| *PKM2* | pyruvate kinase, muscle | aerobic glycolysis |
| *SLC2A4* | solute carrier family 2 (facilitated glucose transporter), member 4 | glucose transport |
| *TPI1* | triosephosphate isomerase 1 | aerobic glycolysis |
| **Cell Differentiation** | | |
| *CES1* | carboxylesterase 1 (monocyte/macrophage serine esterase 1) | adipogenesis |
| *FSTL1* | follistatin-like 1 | cell differenciation |
| *INHBB* | inhibin, beta B | energy balance |
| *MEST* | mesoderm specific transcript homolog (mouse) | adipogenesis |
| *PEX11A* | peroxisomal biogenesis factor 11 alpha | peroxysome organization |
| *ROBO3* | roundabout, axon guidance receptor, homolog 3 (Drosophila) | cell differenciation |
| *SFRP2* | secreted frizzled-related protein 2 | adipogenesis |
| **Cell Proliferation** | | |
| *ANG* | angiogenin, ribonuclease, RNase A family, 5 | angiogenesis |
| *CCND1* | cyclin D1 | cell cycle |
| *CDK2AP1* | cyclin-dependent kinase 2 associated protein 1 | cell cycle |
| *CDKN2C* | cyclin-dependent kinase inhibitor 2C (p18, inhibits CDK4) | cell cycle |
| *DNASE2* | deoxyribonuclease II, lysosomal | apoptosis |
| *FGF2* | fibroblast growth factor 2 (basic) | cell cycle |
| *GHR* | growth hormone receptor | lipolysis |
| *IGF1* | insulin-like growth factor 1 (somatomedin C) | glycogen synthesis |
| *IRS1* | insulin receptor substrate 1 | insulin signaling |
| *IRS2* | insulin receptor substrate 2 | insulin signaling |
| *MAPK3* | mitogen-activated protein kinase 3 | cell cycle |
| *PRKAR2B* | protein kinase, cAMP-dependent, regulatory, type II, beta | signal transduction |
| *VEGFA* | vascular endothelial growth factor A | angiogenesis |
| **Cell Structure** | | |
| *ARPC1A* | actin related protein 2/3 complex, subunit 1A, 41kDa | cytoskeleton organization |
| *CFL1* | cofilin 1 (non-muscle) | cytoskeleton organization |
| *GIT2* | G protein-coupled receptor kinase interacting ArfGAP 2 | cytoskeleton organization |
| *SNTB2* | syntrophin, beta 2 (dystrophin-associated protein A1, 59kDa, basic component 2) | cytoskeleton organization |
| *SPTAN1* | spectrin, alpha, non-erythrocytic 1 (alpha-fodrin) | cytoskeleton organization |
| *TUBA1A* | tubulin, alpha 1a | cytoskeleton organization |
| *TPM3* | tropomyosin 3 | cytoskeleton organization |
| *WDR1* | WD repeat domain 1 | cytoskeleton organization |
| **Energy Metabolism** | | |
| *ADHFE1* | alcohol dehydrogenase, iron containing, 1 | ketone metabolism |
| *AK2* | adenylate kinase 2 | oxydative phosphorylation |
| *ATP5A1* | ATP synthase, H+ transporting, mitochondrial F1 complex, alpha subunit 1, cardiac muscle | oxydative phosphorylation |
| *ATP8A1* | ATPase, aminophospholipid transporter, class I, type 8A, member 1 | oxydative phosphorylation |
| *CKB* | creatine kinase, brain | creatin phosphate shuttle |
| *COX7C* | cytochrome c oxidase subunit VIIc | oxydative phosphorylation |
| *CYCS* | cytochrome c, somatic | oxydative phosphorylation |
| *ETFA* | electron-transfer-flavoprotein, alpha polypeptide | fatty acid oxidation |
| *ETFDH* | electron-transferring-flavoprotein dehydrogenase | fatty acid oxidation |
| *GATM* | glycine amidinotransferase (L-arginine:glycine amidinotransferase) | creatin biosynthesis |
| *IDH1* | isocitrate dehydrogenase 1 (NADP+), soluble | tricarboxylic acid cycle |
| *LEP* | leptin | food intake control |
| *MDH2* | malate dehydrogenase 2, NAD (mitochondrial) | tricarboxylic acid cycle |
| *ME1* | malic enzyme 1, NADP(+)-dependent, cytosolic | tricarboxylic acid cycle |
| *NDUFA9* | NADH dehydrogenase (ubiquinone) 1 alpha subcomplex, 9, 39kDa | oxydative phosphorylation |
| *NDUFB8* | NADH dehydrogenase (ubiquinone) 1 beta subcomplex, 8, 19kDa | oxydative phosphorylation |
| *UCN* | Urocortin | corticoids metabolism |
| *UQCRC2* | ubiquinol-cytochrome c reductase core protein II | oxydative phosphorylation |
| **Immune Response** | | |
| *C1QA* | complement component 1, q subcomponent, A chain | complement system |
| *C1QB* | complement component 1, q subcomponent, B chain | complement system |
| *C1QC* | complement component 1, q subcomponent, C chain | complement system |
| *C2* | complement component 2 | complement system |
| *C3AR1* | complement component 3a receptor 1 | complement system |
| *CCL18* | chemokine (C-C motif) ligand 18 (pulmonary and activation-regulated) | chemotaxis |
| *CCL19* | chemokine (C-C motif) ligand 19 | immune response |
| *CCL2* | chemokine (C-C motif) ligand 2 | chemotaxis |
| *CCL3* | chemokine (C-C motif) ligand 3 | chemotaxis |
| *CCR1* | chemokine (C-C motif) receptor 1 | immune response |
| *CD14* | CD14 molecule | response to LPS |
| *CD163* | CD163 molecule | acute phase response |
| *CD163L1* | CD163 molecule-like 1 | scavenger receptor activity |
| *CD209* | CD209 molecule | cell adhesion |
| *CD48* | CD48 molecule | Ig mediated immune response |
| *CD53* | CD53 molecule | cell adhesion |
| *CD68* | CD68 molecule | scavenger receptor activity |
| *CD74* | CD74 molecule, major histocompatibility complex, class II invariant chain | antigen presentation |
| *CD9* | CD9 molecule | immune response |
| *CD97* | CD97 molecule | cell adhesion |
| *CLEC10A* | C-type lectin domain family 10, member A | cell adhesion |
| *CSF1R* | colony stimulating factor 1 receptor | cell differentiation |
| *FCER1G* | Fc fragment of IgE, high affinity I, receptor for; gamma polypeptide | Ig mediated immune response |
| *FCGBP* | Fc fragment of IgG binding protein | Ig mediated immune response |
| *FCGR2B* | Fc fragment of IgG, low affinity IIb, receptor (CD32) | Ig mediated immune response |
| *FCGRT* | Fc fragment of IgG, receptor, transporter, alpha | Ig transport |
| *FN1* | fibronectin 1 | cell adhesion |
| *FTH1* | ferritin, heavy polypeptide 1 | iron homeostasis |
| *FTL* | ferritin, light polypeptide | iron homeostasis |
| *HLA-A* | major histocompatibility complex, class I, A | antigen presentation |
| *HMOX1* | heme oxygenase (decycling) 1 | hemoglobin degradation |
| *HP* | haptoglobin | hemoglobin degradation |
| *IFI30* | interferon, gamma-inducible protein 30 | antigen presentation |
| *IL10* | interleukin 10 | inflammatory cytokine |
| *IL10RA* | interleukin 10 receptor, alpha | inflammatory response |
| *IL1RN* | interleukin 1 receptor antagonist | inflammatory response |
| *IL4R* | interleukin 4 receptor | inflammatory response |
| *ITGB2* | integrin, beta 2 (complement component 3 receptor 3 and 4 subunit) | cell adhesion |
| *ITGB5* | integrin, beta 5 | cell adhesion |
| *ITGAM* | integrin, alpha M (complement component 3 receptor 3 subunit) | cell adhesion |
| *ITGAX* | integrin, alpha X (complement component 3 receptor 4 subunit) | cell adhesion |
| *KIT* | v-kit Hardy-Zuckerman 4 feline sarcoma viral oncogene homolog | immune response |
| *LCP* | lymphocyte cytosolic protein 1 (L-plastin) | T-cell activation |
| *LILRA6* | leukocyte immunoglobulin-like receptor, subfamily A (with TM domain), member 6 | antigen presentation |
| *LILRB3* | leukocyte immunoglobulin-like receptor, subfamily B (with TM and ITIM domains), member 3 | inflammatory response |
| *LY86* | lymphocyte antigen 86 | inflammatory response |
| *LY96* | lymphocyte antigen 96 | innate immune response |
| *MARCO* | macrophage receptor with collagenous structure | innate immune response |
| *MNDA* | myeloid cell nuclear differentiation antigen | inflammatory response |
| *MRC1L1* | mannose receptor, C type 1-like 1 | receptor-mediated endocytosis |
| *MS4A4A* | membrane-spanning 4-domains, subfamily A, member 4 | inflammatory response |
| *MS4A6A* | membrane-spanning 4-domains, subfamily A, member 6A | macrophage marker |
| *MS4A7* | membrane-spanning 4-domains, subfamily A, member 7 | macrophage marker |
| *MYD88* | myeloid differentiation primary response gene (88) | inflammatory response |
| *NFKB2* | nuclear factor of kappa light polypeptide gene enhancer in B-cells 2 (p49/p100) | transcription |
| *PLA2G7* | phospholipase A2, group VII (platelet-activating factor acetylhydrolase, plasma) | inflammatory response |
| *RAC1* | ras-related C3 botulinum toxin substrate 1 (rho family, small GTP binding protein Rac1) | immune response |
| *S100A4* | S100 calcium binding protein A4 | inflammatory response |
| *SAA4* | serum amyloid A4, constitutive | acute phase response |
| *SCARA5* | scavenger receptor class A, member 5 (putative) | iron transport |
| *SPP1* | secreted phosphoprotein 1 | chemotaxis |
| *TFRC* | transferrin receptor (p90, CD71) | iron homeostasis |
| *THBS4* | thrombospondin 4 | cell adhesion |
| **Lipid Metabolism** | | |
| *AACS* | acetoacetyl-CoA synthetase | lipogenesis |
| *AADACL1* | neutral cholesterol ester hydrolase 1 | cholesterol metabolism |
| *ABHD5* | abhydrolase domain containing 5 | lipolysis |
| *ACACB* | acetyl-Coenzyme A carboxylase beta | lipogenesis |
| *ACAD9* | acyl-Coenzyme A dehydrogenase family, member 9 | lipogenesis |
| *ACADM* | acyl-Coenzyme A dehydrogenase, C-4 to C-12 straight chain | fatty acid oxydation |
| *ACAT1* | acetyl-Coenzyme A acetyltransferase 1 | lipogenesis |
| *ACOX1* | acyl-Coenzyme A oxidase 1, palmitoyl | fatty acid oxydation |
| *ACSL1* | acyl-CoA synthetase long-chain family member 1 | lipogenesis |
| *ACSS2* | acyl-CoA synthetase short-chain family member 2 | lipogenesis |
| *AGPAT1* | 1-acylglycerol-3-phosphate O-acyltransferase 1 (lysophosphatidic acid acyltransferase, alpha) | lipogenesis |
| *AGPAT9* | 1-acylglycerol-3-phosphate O-acyltransferase 9 | lipogenesis |
| *ALOX12* | arachidonate 12-lipoxygenase | leukotriene synthesis |
| *ALOX5* | arachidonate 5-lipoxygenase | leukotriene synthesis |
| *AQP1* | aquaporin 1 (Colton blood group) | glycerol transporter |
| *AQP7* | aquaporin 7 | glycerol transporter |
| *AZGP1* | alpha-2-glycoprotein 1, zinc-binding | lipolysis |
| *CIDEA* | cell death-inducing DFFA-like effector a | fatty acid oxydation |
| *CIDEC* | cell death-inducing DFFA-like effector c | lipogenesis |
| *DCI* | dodecenoyl-Coenzyme A delta isomerase (3,2 trans-enoyl-Coenzyme A isomerase) | fatty acid oxydation |
| *DGAT1* | diacylglycerol O-acyltransferase homolog 1 (mouse) | lipogenesis |
| *DGAT2* | diacylglycerol O-acyltransferase homolog 2 (mouse) | lipogenesis |
| *ECHDC1* | enoyl Coenzyme A hydratase domain containing 1 | fatty acid oxydation |
| *ECHDC3* | enoyl Coenzyme A hydratase domain containing 3 | fatty acid oxydation |
| *ELOVL5* | ELOVL family member 5, elongation of long chain fatty acids (FEN1/Elo2, SUR4/Elo3-like, yeast) | lipogenesis |
| *FABP4* | fatty acid binding protein 4, adipocyte | fatty acid transport |
| *FADS1* | fatty acid desaturase 1 | lipogenesis |
| *FADS2* | fatty acid desaturase 2 | lipogenesis |
| *FASN* | fatty acid synthase | lipogenesis |
| *GPD1L* | glycerol-3-phosphate dehydrogenase 1-like | phospholipid metabolism |
| *GPR109A* | G protein-coupled receptor 109A | lipolysis |
| *HADH* | Hydroxyacyl-Coenzyme A dehydrogenase | fatty acid oxidation |
| *HSDL2* | hydroxysteroid dehydrogenase like 2 | lipolysis |
| *KLB* | klotho beta | cholesterol metabolism |
| *LASS2* | LAG1 homolog, ceramide synthase 2 | sphingolipid metabolism |
| *LDLR* | low density lipoprotein receptor | cholesterol metabolism |
| *LIPA* | lipase A, lysosomal acid, cholesterol esterase | cholesterol metabolism |
| *LIPE* | lipase, hormone-sensitive | lipolysis |
| *LPCAT1* | lysophosphatidylcholine acyltransferase 1 | phospholipid synthesis |
| *LPIN1* | lipin 1 | lipogenesis |
| *MECR* | mitochondrial trans-2-enoyl-CoA reductase | fatty acid oxydation |
| *OSBPL9* | oxysterol binding protein-like 9 | cholesterol transport |
| *PCCA* | propionyl Coenzyme A carboxylase, alpha polypeptide | fatty acid oxidation |
| *PECI* | peroxisomal D3,D2-enoyl-CoA isomerase | fatty acid oxidation |
| *PECR* | peroxisomal trans-2-enoyl-CoA reductase | fatty acid biosynthesis |
| *PGDS* | prostaglandin-H2 D-isomerase | prostaglandin metabolism |
| *PHYH* | phytanoyl-CoA 2-hydroxylase | fatty acid oxidation |
| *PKIG* | protein kinase (cAMP-dependent, catalytic) inhibitor gamma | lipolysis |
| *PNPLA2* | patatin-like phospholipase domain containing 2 (ATGL) | lipolysis |
| *PNPLA3* | patatin-like phospholipase domain containing 3 (adiponutrin) | lipolysis |
| *RDH10* | retinol dehydrogenase 10 (all-trans) | retinol metabolism |
| *SCD* | stearoyl-CoA desaturase (delta-9-desaturase) | lipogenesis |
| *THRSP* | thyroid hormone responsive (SPOT14 homolog, rat) | lipogenesis |
| *WISP2* | WNT1 inducible signaling pathway protein 2 | adipogenesis |
| **Protein Metabolism** | | |
| *CST3* | cystatin C | tissue remodelling |
| *CSTB* | cystatin B (stefin B) | tissue remodelling |
| *CTSB* | cathepsin B | tissue remodelling |
| *CTSC* | cathepsin C | proteolysis |
| *CTSZ* | cathepsin S | proteolysis |
| *CTSZ* | cathepsin Z | tissue remodelling |
| *EMILIN2* | elastin microfibril interfacer 2 | tissue remodelling |
| *LOX* | lysyl oxidase | tissue remodelling |
| *LOXL1* | lysyl oxidase-like 1 | tissue remodelling |
| *LOXL2* | lysyl oxidase-like 2 | tissue remodelling |
| *MMP19* | matrix metallopeptidase 19 | tissue remodelling |
| *MMP9* | matrix metallopeptidase 9 (gelatinase B, 92kDa gelatinase, 92kDa type IV collagenase) | tissue remodelling |
| *PLAU* | plasminogen activator, urokinase | proteolysis |
| *PLAUR* | plasminogen activator, urokinase receptor | proteolysis |
| *PSMC4* | proteasome (prosome, macropain) 26S subunit, ATPase, 4 | proteolysis |
| *RNF5* | ring finger protein 5 | proteolysis |
| *RPN1* | ribophorin I | protein glycosylation |
| *TPST2* | tyrosylprotein sulfotransferase 2 | peptidyl-tyrosine sulfation |
| **Response to Stress** | | |
| *ATOX1* | ATX1 antioxidant protein 1 homolog (yeast) | copper ion transport |
| *MT1E* | metallothionein 1E | response to oxidative stress |
| *MT1G* | metallothionein 1G | response to oxidative stress |
| *PRDX6* | peroxiredoxin 6 | response to oxidative stress |
| *OXSR1* | oxidative-stress responsive 1 | response to oxidative stress |
| **Signal Transduction** | | |
| *ACTR3* | ARP3 actin-related protein 3 homolog (yeast) | endocytosis signaling |
| *AP2M1* | adaptor-related protein complex 2, mu 1 subunit | intracellular trafficking |
| *EHD4* | EH-domain containing 4 | endocytosis signaling |
| *LGR4* | leucine-rich repeat-containing G protein-coupled receptor 4 | membrane receptor |
| *MAOA* | monoamine oxidase A | dopamin catabolism |
| *SNCA* | synuclein, alpha (non A4 component of amyloid precursor) | membrane trafficking |
| **Gene Expression** | | |
| *AES* | amino-terminal enhancer of split | transcription |
| *ATF3* | activating transcription factor 3 | transcription |
| *CARHSP1* | calcium regulated heat stable protein 1, 24kDa | translation |
| *E2F4* | E2F transcription factor 4, p107/p130-binding | transcription |
| *EIF2B1* | eukaryotic translation initiation factor 2B, subunit 1 alpha, 26kDa | translation |
| *EIF4A1* | eukaryotic translation initiation factor 4A1 | translation |
| *EN2* | engrailed homeobox 2 | transcription |
| *IRF5* | interferon regulatory factor 5 | transcription |
| *MCM3* | minichromosome maintenance complex component 3 | transcription |
| *MSH6* | mutS homolog 6 (E. coli) | transcription |
| *NEUROG3* | neurogenin 3 | transcription |
| *NPAS3* | neuronal PAS domain protein 3 | transcription |
| *NRIP1* | nuclear receptor interacting protein 1 | transcription |
| *PELP1* | proline, glutamate and leucine rich protein 1 | transcription |
| *PWP1* | PWP1 homolog (S. cerevisiae) | transcription |
| *RNH1* | ribonuclease/angiogenin inhibitor 1 | transcription |
| *SREBF1* | sterol regulatory element binding transcription factor 1 | transcription |
| *SIRT1* | sirtuin (silent mating type information regulation 2 homolog) 1 (S. cerevisiae) | histone deacetylase |
| *SRP9* | signal recognition particle 9kDa | translation |
| *TCEAL8* | transcription elongation factor A (SII)-like 8 | transcription |
| *TDRD7* | tudor domain containing 7 | translation |
| *TNRC6B* | triosephosphate isomerase 1 | translation |
| *TSEN54* | tRNA splicing endonuclease 54 homolog (S. cerevisiae) | translation |
| *TWIST1* | twist homolog 1 (Drosophila) | transcription |
| *VGLL3* | vestigial like 3 (Drosophila) | transcription |
| **Transport** | | |
| *ARF3* | ADP-ribosylation factor 3 | protein trafficking |
| *EXOC1* | exocyst complex component 1 | exocytosis |
| *LAPTM5* | lysosomal protein transmembrane 5 | lysosome transport |
| *NUP62* | nucleoporin 62kDa | mRNA transport |
| *CD52* | CD52 molecule | carbohydrates transport |
| *POP4* | processing of precursor 4, ribonuclease P/MRP subunit (S. cerevisiae) | RNA transport |
| *SLC19A2* | solute carrier family 19 (thiamine transporter), member 2 | thiamine transport |
| *SLC19A3* | solute carrier family 19, member 3 | thiamine transport |
| *SLC35C2* | solute carrier family 35, member C2 | response to hypoxia |
| *SLC4A4* | solute carrier family 4, sodium bicarbonate cotransporter, member 4 | Na-HCO3 transport |
| *TXNDC5* | thioredoxin domain containing 5 (endoplasmic reticulum) | vesicule-mediated transport |
| **Miscellaneous** | | |
| *BTBD7* | BTB (POZ) domain containing 7 | unknown |
| *CIDECP* | cell death-inducing DFFA-like effector c pseudogene | unknown |
| *CYYR1* | cysteine/tyrosine-rich 1 | unknown |
| *DNAJC13* | DnaJ (Hsp40) homolog, subfamily C, member 13 | unknown |
| *NOMO1* | NODAL modulator 1 | unknown |
| *SH3BGRL* | SH3 domain binding glutamic acid-rich protein like | unknown |
| *TMEM135* | transmembrane protein 135 | unknown |
