# Supplementary material for: Adipose Tissue CIDEA Is Associated, Independently of Weight Variation, to Change in Insulin Resistance during a Longitudinal Weight Control Dietary Program in Obese Individuals
Source: PLoS One. 2014 Jul 1;9(7):e98707. doi: 10.1371/journal.pone.0098707 (PMC4077708; doi:10.1371/journal.pone.0098707)
Supplement: Protocol S1 — Detailed study protocol of the DiOGenes trial. (PDF) [file pone.0098707.s002.pdf]

Study: Diet, Obesity and Genes (DiOGenes)

Title: A randomised controlled trial comparing the effect of reduced-fat diets varying in glycaemic index (high vs. low) and protein content (high vs. normal) on bodyweight in overweight and obese subjects after an initial weight loss

Location:
The Department of Human Nutrition, The Royal Veterinary and Agricultural University (RVAU), Rolighedsvej 30, DK-1958 Frederiksberg, Denmark.

STUDY PROTOCOL

This study is part of the EU-sponsored project DiOGenes (1), which is funded by the EU Sixth Framework Programme under Priority 5: Food Quality and Safety (Web-site: www.diogenes-eu.org) (FP6-513946). DiOGenes comprises 5 strategic Research, Technological and Development (RTD) lines, each with its specific objectives. This protocol describes the dietary intervention study which is to be carried out by RTD line 1 (Obesity and macronutrient composition of the diet).

1. SYNOPSIS

Study
DiOGenes is a randomised controlled dietary intervention study in obese/overweight families (adults and children) in 8 different European countries, testing the efficacy and safety of diets differing in Glycaemic Index (GI) and protein content.

Aims
The main objective of the DiOGenes study is to identify the efficacy of particular diets for protection against weight gain and weight regain, in a susceptible population of obese and overweight individuals and their children. The overall study comprises a 6 to 12 months dietary intervention study in 350 families in 8 European countries (Denmark, the Netherlands, UK, Germany, Spain, Greece, Bulgaria and the Czech Republic), investigating the effect of different dietary components (high/normal protein and high/low GI) on maintenance of weight loss and prevention of weight (re)gain. In Denmark/Netherlands the study will be carried out over 12 months, in about 50 families. The responsiveness of the families to different dietary compositions and effects on body weight control and obesity related risk factors will be assessed during the dietary intervention study, with specific focus on changes in body weight and composition, appetite measures, adipose tissue gene expression and risk factors for cardiovascular disease (CVD) and type-2 diabetes. The role of genetic, environmental, social and cultural factors as well as their interactions with the studied diets on body weight control or development of obesity will also be investigated.

Primary outcome measures for adults are body weight loss maintained (kg), change in body composition, proportion of subjects maintaining > 0, 5 and 10% weight loss, and drop-out rate. Other non-clinical primary measures are to identify biological and psychological traits that determine the family's and individual's responses to the dietary interventions. Secondary end-points are reduction in abdominal obesity, changes in risk factors of type-2 diabetes and CVD and appetite/satiety hormones, and changes in physical activity. Clinical measurements will include in addition the identification and quantification of adipose tissue mRNA and peptide biomarkers.
Primary outcome measures for children are changes in the proportion of overweight and obese children at 6 mo, and changes in BMI, and % whole body fat. Secondary end-points for overweight children are reduction in abdominal obesity, changes in waist-hip circumference ratio and blood parameters and changes in physical activity. A sub-study (described below) will assess the effect and safety of the high protein diet in children and adolescents. Dietary effects on bone and kidney health will be assessed and primary safety end-points will include changes in urinary and blood proteins, such as albumin and creatinine.

Subjects
Families where both parents are < 65 years old, and where at least one parent is obese (BMI>30 kg/m^2) and the other is overweight (BMI>28 kg/m^2) will be offered inclusion in the study with the aim of losing weight and maintaining the weight loss. Families will have 1-3 children, and at least one child aged between 5 and 18 years will be overweight (defined according to Cole *et al.* (2), i.e. with an iso-BMI>25). A total of 150 adult subjects plus children (75 families) will be screened and approximately 135 adults are expected to be included in the initial weight loss phase of the study, with the aim of having a total of 110 subjects for the subsequent randomization to the 5 dietary

arms. This number will provide an anticipated completion of more than 80 subjects at the 12-month visit.

Subjects will be recruited through the department's waiting list, local press or via referrals from local general practices or from other medical departments. Radio/television or newspaper advertising may also be employed if necessary. Eligible subjects will only be included after they have been informed about the study and signed informed consent forms (including children, see Section 11).

Inclusion criteria

Families with 1 obese (BMI>30 kg/m^2) and 1 overweight (BMI>28 kg/m^2) parent, with 1-3 children, one of whom must be aged 5-18 and overweight (iso-BMI>25). Single-parent families with one obese (BMI>30 kg/m^2) parent and minimum 1 overweight child aged 5-18 will also be included.

Exclusion criteria

Adults: Body weight changes more than ± 3 kg within the last 2 months, pregnant or lactating women, subjects with heart or coronary diseases, kidney or liver diseases, psychiatric illnesses, systemic infections or endocrine diseases, a history of malabsorption, subjects with any clinical condition that renders them unfit to participate, hypertensive and/or hypercholesterolaemic subjects with pharmacological changes within the last 2 months, systolic blood pressure (SBP) > 160 and/or diastolic blood pressure (DBP) > 100 mmHg, subjects on special diets.
Children: Subjects with systemic infections or chronic disease, or on special diets.

Dietary periods

1) Adult subjects only: 8 week Low Calorie Diet (LCD) period (liquid formula diet, 800-1000 kcal/day).
2) 6 months dietary intervention period 1 (SUPERMARKET period) followed by
3) 6 months dietary intervention period 2 (DIETARY INSTRUCTION period).
4) 12 months free-living period with no dietary intervention.

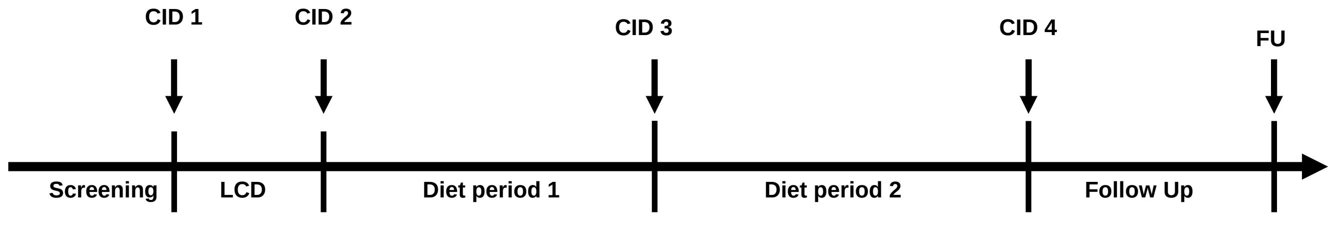

Diets and randomization

Families will be randomised into 5 dietary groups during dietary intervention periods 1 and 2:

Group 1: Normal protein/low GI
Group 2: Normal protein/high GI
Group 3: High protein/low GI
Group 4: High protein/high GI
Group 5: Control diet

The eligible families will be stratified based on centre, number of obese parents and BMI. Within the strata the subjects will be randomized to treatment (and sub-study groups) using a simple block randomization method by a third party having no knowledge of the subjects that may affect the outcome of the study.

Dietary intervention study

All adult subjects will for the first 8 weeks of the study be submitted to an 800-1000 kcal/day LCD diet combined with instructions on behaviour modification. Only families where at least 1 parent has achieved a >8% weight loss will be included in the intervention study. The study consists of a 6-month dietary intervention period (period 1, SUPERMARKET period) where families obtain free food from an on-site supermarket and receive continual dietetic guidance, followed by a further 6-month period (period 2, DIETARY INSTRUCTION period) where families receive dietary and behavioural instruction. Families will regularly visit RVAU/UM throughout the intervention period for weighing and dietary instruction. There are 4 Clinical Investigation Days (CIDs) planned during the study, when anthropometric and other measures will be taken. CID 1 will take place in the 3 weeks prior to the LCD diet. CID 2[1], 3 and 4 will take place in weeks 0, 26 and 52, respectively.

Intervention outcome measures

During the intervention study, questionnaires and 3-day food records will be completed by all adult subjects, and fat biopsies and fasting blood samples will be taken, anthropometric measurements (weight, body composition, etc) and 24h urine collections will be made and oral glucose tolerance tests (OGTT) will be performed. Some adult subjects are involved in specific sub-studies (described in Section 6).

The overweight children (aged 5 - 18) will also complete 3-day dietary records, and will have blood and urine samples taken (but not fat biopsies) for measurement of safety factors and certain hormones, and will also undergo anthropometric measurements (height, weight etc). Normal weight children will undergo anthropometric measurements and have blood and urine samples taken for measurement of safety factors only. All these measures and the visits planned are described in more detail ahead (Section 9).

Compliance monitoring procedures

Before, during and after the intervention, compliance assessment is carried out to evaluate and ensure adherence to the dietary regimens. In addition to self-reported dietary records (adults and children), blood and urinary samples will be taken (adults only) to assure and monitor the adherence to the dietary regimens.

Duration of the study

The study will begin recruitment of families by September 2005 and will start in January 2006 (LCD diet), with a staggering period of about 12 weeks. The 12 months intervention period will be finished in around June 1, 2007 and the final 24-month follow up visit will complete the study in around June 2008.

Ethics

The study will be performed according to the latest version of the Declaration of Helsinki, the UN Convention on the Rights of the Child and the Current International Conference on Harmonization

[1] CID 2 for children will take place in week 4

(ICH) guidelines. Wherever possible, the study will be performed according to the current Good Clinical Practice (GCP) guidelines. An application will be submitted to the regional Ethics Committee and the study will not be undertaken without a positive statement from the committee regarding the study. All subjects (including children where possible) must give verbal and written informed consent before they can be entered into the study.

Insurance

The subjects will be insured via the public patient insurance. 2. LIST OF ABBREVIATIONS

AE	Adverse Event
ANACOVA	Analysis of Covariance
BP	Blood Pressure
BMC	Bone Mineral Content
BMI	Body Mass Index
BMR	Basal Metabolic Rate
CID	Clinical Investigation Day
CVD	Cardiovascular Disease
DBP	Diastolic Blood Pressure
DiOGenes	Diet, Obesity and Genes
DEXA	Dual-energy X-ray Absorptiometry
DLW	Doubly labelled water
FM	Fat Mass
GCP	Good Clinical Practice
GI	Glycaemic Index
ICH	International Conference on Harmonization
IGF-1	Insulin-like Growth Factor-1
IGFBP-3	IGF Binding Protein-3
LCD	Low Calorie Diet
LTM	Lean Tissue Mass
OGTT	Oral Glucose Tolerance Test
RDA	Recommended Daily Allowance
RTD	Research, Technological and Development
RVAU	Royal Veterinary and Agricultural University
SAE	Serious Adverse Event
SBP	Systolic Blood Pressure
SD	Standard Deviation
TBW	Total Body Weight
VAS	Visual Analogue Scales

2. BACKGROUND

Obesity is generally regarded to be the most prevalent nutritional disorder in Europe. As the prevalence of obesity has increased, so have the number of clinical and scientific studies aimed at determining which dietary components are most efficacious for promoting and sustaining weight loss. A large body of evidence has demonstrated that a reduction in dietary fat content under *ad libitum* conditions can produce a modest but clinically important weight loss in 6-12 months (3), and in combination with a slight increase in physical activity can prevent 58% of new cases of type 2 diabetes in high risk obese subjects (4). It is clear, however, that the non-fat component of the diet is also critically important.

The glycaemic index (GI) of carbohydrates has been demonstrated to have importance for cardiovascular risk factors and glycaemic control in diabetics, and has been suggested to play a role in appetite control (5). A number of studies, and several popular diet books, attribute passive over-consumption of energy to a diet with a high GI, and suggest that replacing high GI foods with similar low GI foods can prevent this or lead to a greater loss of fat compared to lean body tissue (5;6). The literature, however, is conflicting. Systematic reviews are equivocal and point to many negative studies that suffer from lack of dietary compliance and standardisation, and lack of statistical power to detect weight changes of clinical relevance and particularly of public health relevance (7;8). It is also pertinent to note that even the concept of GI has been questioned because of its poor reproducibility (GI is affected by fruit ripeness, food particle size, type of a particular food, food processing and cooking methods, for instance) (8). Furthermore, most GI studies to date are of short duration and have been carried out with single food items as opposed to mixed meals. The current study will take these shortcomings into consideration.

Ad libitum diets with high protein content have been shown to enhance satiety (9;10) and increase thermogenesis (11) in a number of smaller studies, and also produce better weight control and weight loss than diets in which the protein is replaced by carbohydrate (12;13). These high-protein diets are challenging the current recommendations (14) and need to be evaluated for efficacy and safety in a large, long-term, strictly controlled intervention trial (13). The Institute of Medicine (15) has recently increased the recommended acceptable daily protein intake for adults to 10-35% caloric intake and for children (aged 4-18) to10-30%. A previous 6-month study found that a fat-reduced diet with high compared to normal protein enhanced weight loss and provided a greater reduction in intra-abdominal fat stores (12). However this pilot study did not have the statistical power to assess weight differences between groups after 12 months due to inadequate numbers of subjects (16). Moreover, adverse effects of high protein diets on renal function in healthy individuals have not been clearly established to date, and effects on bone are unclear (13). The safety and tolerability of a high protein diet in different age groups thus requires clarification, in particular in children; this will be exerted in the current study.

The rapid increase in childhood obesity which currently affects all European countries is of particular societal concern today. This study is novel because it focuses on the whole family. The aim is to identify the diet most effective for protection against weight gain and weight regain in a susceptible population of obese and overweight individuals and their children. Investigating the diet within the whole family should encourage overweight children to lose weight; indeed some studies have demonstrated that the family based approach to body weight management is most effective for weight loss. In addition the diet most beneficial for risk factors of CVD and type-2 diabetes also

requires clarification (5;14). The uncertainty about the optimal diet composition for the prevention and treatment of obesity is a public health problem which needs to be addressed by a large long-term randomized intervention trial. This is the background for the proposed dietary intervention study, which hopefully will contribute to the knowledge in this area and provide valid evidence that may help decision makers to make appropriate dietary recommendations.

3. DESIGN

This randomised controlled dietary intervention study will involve approximately 50 families in Denmark (75 initially screened, thus allowing for drop-out). Families will comprise at least 1 obese parent (BMI>30 kg/m^2) and 1 overweight(2) child (iso-BMI>25); the other parent will be overweight (BMI>28 kg/m^2). After screening (see Sections 7.1 & 7.2), all adult subjects will initially undergo an 8-week LCD weight loss diet. This will be followed by a 12 months *ad libitum* dietary intervention study (see below), consisting of a 6-month SUPERMARKET period and a 6-month DIETARY INSTRUCTION period. Subsequently there will be a 24-month follow-up after 12 months free-living with no dietary intervention (see Study Flow Diagram, Section 2).

CID 1 will take place in the 3 weeks prior to the LCD diet. This will include (adults) anthropometric measurements, BP, blood sample and 24-h urine collection, fat biopsy, OGTT and analysis of body composition using dual-energy x-ray absorptiometry (DEXA), and completion of lifestyle/behavioural questionnaires. For all children, CID 1 will include anthropometric measurements and blood sample/urine collection for safety measures, with additional measures of body composition (DEXA/bioimpedance) and blood samples for hormone analysis for overweight children only.

A number of subgroup investigations (adults only) will also be initiated in the 3 weeks prior to the LCD diet:
Subgroup a: This subgroup will complete a 7-day food record and undergo basal metabolic rate (BMR) and doubly labelled water (DLW) measurements, have physical activity measured by use of a pedometer and have a meal test.
Subgroup b: This sub-group will have one extra fat-biopsy taken on the 3rd day into the LCD diet.

1. 8 week LCD dietary period

The energy restricted LCD diet (for adults only) will consist of 800-1000 kcal/d and the target macronutrient composition of the diet will be 15-20% of total energy from fat, 35-40% from protein and 45-50% from carbohydrate. No energy restriction will be imposed for the children in the enrolled families. During this period, subjects will attend approx. 6 group meetings at the Department where their body weight, adverse events (AE) and concomitant medication will be registered and dietary and behavioural instructions will be given.

Before entering intervention period 1, adult subjects visit the Department for CID 2. This will again include anthropometric measurements, BP, blood sample and 24-h urine collection, fat biopsy, OGTT, analysis of body composition (DEXA scan), and completion of lifestyle/behavioural questionnaires. Families where at least one parent achieves a >8% weight loss will be randomised to one of the 5 dietary arms.

Subgroup a will complete a 7-day food record and undergo BMR and DLW measurements, have physical activity measured by use of a pedometer and have a meal test.

2. 6 months dietary intervention period 1 (SUPERMARKET period)

Families in which at least one parent has achieved an 8% loss of initial body weight will be randomised to one of 5 dietary arms (see Table 1), including a control group. Subjects enrolled in the 4 intervention groups will be expected to get most of their foods free of charge in the supermarket for the first 6 months of the intervention. The on-site supermarket has fresh, shelved and frozen products, separated according to the dietary design. All food items will be bar-coded and a dietician will scan the barcodes of all the chosen items to monitor macronutrient composition of the diet and will assist in altering the selection to meet the prescribed macronutrient composition. Each food item scanned will be registered in a database in the shop. The calculated energy content of the chosen groceries will not be made known to the subjects. The control group will be instructed to continue their habitual dietary intake whilst shopping in ordinary food shops (thus their food will not be free).

Families in all intervention groups will receive careful, intensive and regular dietary and behavioural guidance during both intervention periods 1 and 2, although the control group will not receive advice regarding dietary composition. The dietary instruction will include family sessions, where instruction will be given to the whole family, including children. The dietician will advise on weight control and reinforce the diet composition through recipes, cooking advice, and behaviour modification in group (several families) and individual meetings (one family). Subjects will be given oral and written instructions relating to the five intervention groups based on an exchange system. The aim is to reach the required macronutrient composition on a daily basis. Hence, individual meals (breakfast, lunch, dinner) may not necessarily have the designated macronutrient composition. During intervention periods 1 and 2, weight maintenance is attempted, though further weight reduction will be allowed, i.e. in all 5 groups, the diet intervention is carried out with less emphasis on the calorie content of the diet (which is *ad libitum*) or the calorie density of the diet, but with major emphasis on adherence to the diet prescribed. Hence, during both periods 1 and 2 it is the diets' ability to satisfy and to regulate appetite and body weight that will be tested.

In week 4 of period 1, the children will visit the Department for their CID 2. This will include (all children) anthropometric measurements and blood sample/urine collection, with additional measures of body composition (DEXA/bioimpedance) for overweight children only.

Before entering intervention period 2, adults and children visit the Department for CID 3, including (adults) anthropometric measurements, BP, blood sample and 24-h urine collection, fat biopsy, OGTT and analysis of body composition (DEXA scan). Measures for children include (all children) anthropometric measurements and blood sample/urine collection, with additional measures of body composition (DEXA/bioimpedance) for overweight children only.

Subgroup a will complete a 7-day food record and undergo BMR and DLW measurements, and have physical activity measured by use of a pedometer.

3. 6 months dietary intervention period 2 (DIETARY INSTRUCTION period)

Families will be followed for another 6-month intervention period (Period 2), although free food will not be supplied and families will be expected to shop in ordinary supermarkets. Throughout this period the subjects will again receive dietary/behavioural instructions. Families will be encouraged to follow the dietary regimens in accordance with their randomization. Also during this phase, weight maintenance is attempted, though further weight reduction will be allowed.
At the end of intervention period 2, CID 4 will take place. This will include (adults) anthropometric measurements, BP, blood sample and 24-h urine collection, OGTT and analysis of body composition (DEXA scan). For all children, CID 4 will include anthropometric measurements and blood sample/urine collection, with additional measures of body composition (DEXA/bioimpedance) for overweight children only.

4.1 DIETARY GROUPS

Table 1: Dietary groups during the intervention study.

	Low glycaemic index	High glycaemic index
Normal protein	1	2
High protein	3	4
Control	5	

1. 25-30% of energy from fat, 10-15% from protein and 57-62% from low GI carbohydrates.
2. 25-30% of energy from fat, 10-15% from protein and 57-62% from high GI carbohydrates.
3. 25-30% of energy from fat, 23-28% from protein and 45-50% from low GI carbohydrates.
4. 25-30% of energy from fat, 23-28% from protein and 45-50% from high GI carbohydrates.
5. Control diet, concordant with the family's habitual diet.

High protein content is defined as 25% of energy intake (range 23-28%); although the long term safety of a high protein diet may not be fully evaluated, these percentages were used in a previous 6-month study of high protein intake(12) and are within the acceptable range for both children (10-30%, children aged 4-18) and adults (10-35%) (15). Thus the lower level for protein of 12% of energy intake (range 10-15%) is also within the normal Recommended Daily Allowance (RDA) range for children and adults (15). The protein sources will be primarily of animal origin, such as lean meat and low fat dairy products, but will also include vegetable proteins, such as pulses (beans, lentils and peas), which are usually quite inexpensive.

High GI is concordant with the habitual European diet (5) but may also be linked with increased risk of obesity, therefore the potential adverse effects of this type of diet will be examined within the study. The glycaemic level of the diet will be determined on the basis of international GI tables (17).

4.2 METHOD OF ASSIGNING FAMILIES TO DIETARY GROUPS

The eligible families will be allocated to a dietary intervention group using a simple block randomization procedure with centre, number of obese parents in the family and BMI (greater or less than 34, screening value) as stratification criteria by a third party with no knowledge of the

subjects that may affect the outcome of the study. Approx. 11 families are randomized to each of the 5 dietary intervention groups. The outcome in the dietary intervention groups will be compared to each other and to the control group.

Approx. 6 subjects in each dietary group (3 men and 3 women) will be randomized to **Subgroup a (**7-day food record, BMR and DLW measurements, physical activity using pedometers, meal test). Approx. 7-10 subjects in each dietary group will be randomized to **Subgroup b** (extra fat biopsy day 3 into LCD diet).
Participation by (adult) subjects in the sub-groups is entirely voluntary and separate information sheets and consent forms will be signed.

4.3 BLINDING

Due to the characteristics of the study design where the dietetic guidance of the study subjects is the inherent intervention in the study, any blinding of the dietician or subjects is not possible. Each dietician will be responsible for guidance of subjects in all five dietary intervention groups.
By any analyses of data obtained prior to study termination, it will be assured that these analyses will not influence the ongoing study and hence the final outcome of the study.
These restrictions will be inferred to exclude any bias introduced by the study personnel.

5. SUBJECTS

An estimated 75 families will undergo screening. Thus it is aimed to enrol a total of about 50 families in the dietary intervention study, allowing for drop-out.

5.1 INCLUSION CRITERIA

The inclusion criteria for the families are as follows:

- Families where both parents are < 65 years old, and where minimum one adult parent is obese (BMI>30) and the other parent is BMI>28.
- Families with 1-3 children, where at least one child aged between 5 and 18 years is overweight (defined according to Cole *et al*. (2), i.e. with an iso-BMI>25).
- Single-parent families with one obese (BMI>30) parent and minimum 1 overweight child aged between 5 and 18 years[2]. Any other adult family members (including offspring) are excluded from the study. Children above the age of 16 may be excluded if they so wish.
- Healthy and no regular medication (exception oral contraceptives)
- All ethnic groups are allowed inclusion
- Smoking is allowed, provided subjects do not intend to change their smoking behaviour.
- All subjects should have an overall physical and psychological condition that the investigator believes is in accordance with the overall aim of the study.

5.2 EXCLUSION CRITERIA

The exclusion criteria for the adults are as follows:

[2] Children below the age of 5 are excluded and should continue to eat according to local dietary guidelines

At enrolment:

- Use of prescription medication marked with N
- Blood pressure above 160/100 mmHg (Subjects with SBP=159 and DBP=99 are allowed inclusion). Well regulated BP (<140/90 mmHg) using drugs is allowed if no change in BP and drug dose within last 3 months.
- Fasting blood glucose >6.1 mM at screening. Re-test is allowed.
- Known untreated hypercholesterolemia (>7 mM). Subjects using drugs for hypercholesterolemia are excluded.
- Known untreated hypertriglyceridemia (>3 mM). Subjects using drugs for hyper triglyceridemia are excluded.
- Drug-treated thyroid diseases (well substituted hypothyroidism is allowed inclusion) [3]
- food allergies
- special diets (e.g. vegetarian, Atkins) within 2 months prior to study start
- Food intolerance expected to interfere with the study
- Regularly drinking > 21 alcoholic units/week (men), or > 14 alcoholic units/week (women)
- Elite athletes and subjects planning to engage in elite sports (i.e. planning major changes in physical activity during the study).
- Blood donation within the past 3 months before entering the study.
- Weight change of > 3 kg within 2 months prior to Clinical Investigation Day
- Mental disorders
- Eating disorders
- Pregnant or lactating women, or women who are planning to become pregnant within the next 18 months.
- Surgically treated obesity
- Participation in other clinical studies within the last 3 months
- Alcohol or drug abuse (based on clinical judgment)
- Subjects who are unable to give informed consent.
- Subjects with systemic infections
- Subjects with endocrine disease
- Subjects with malabsorption
- Subjects who are unable to engage in a 8 week LCD period

At randomisation:

- Families in which both adults fail to lose min. 8% of initial body weight during LCD period

The exclusion criteria for the overweight children are as follows:

- special diets (e.g. vegetarian, Atkins) within 2 months prior to study start
- food intolerance expected to interfere with the study
- Regularly drinking > 21 alcoholic units/week (male), or > 14 alcoholic units/week (female)
- Blood donation within the past 3 months before entering the study.
- Subjects with systemic infections / chronic disease
- Use of chronic medication, except for conditions like hayfever

5.3 PROCEDURE FOR EXCLUSION/DISCONTINUATION DURING THE STUDY

A subject will be removed from the study treatment based on their own decision or if the investigator finds it medically necessary.
If any child or adult refuses to follow dietary instruction after starting the intervention, the other family members will still be allowed to continue in the study. However, if refusal (by adult or overweight child) occurs before the start of the LCD diet, then the family will not be included in the study.
When a subject decides to discontinue participation in the study, he/she should always be contacted in order to, if possible, obtain information about the reason(s) for discontinuation. Whenever possible, the subjects should return for an unscheduled visit at the time of or soon after discontinuation.
The following procedures should be performed at an unscheduled visit:

- Date of discontinuation
- Reason for discontinuation
- Adverse events
- Recording of any new concomitant drug therapy or any changes to ongoing concomitant drug therapy since last visit, any pharmacological treatment established prior to entrance in this study will be maintained as long as ethically possible
- DEXA scan or bioimpedance (body fat mass, body lean mass)
- Body weight
- Blood pressure
- Waist/hip and sagittal measurements
- Blood sample
- Compliance Questionnaire

Subjects may also be withdrawn from the study because of severe protocol violations.
These subjects will not be included in the analysis and will permanently be dismissed from the study. Reasons for dismissal should be carefully recorded in the source data.

6. STUDY TIME SCHEDULE

The study is planned to initiate recruitment of families by September 2005 and to be started in January 2006 (LCD diet), with a staggering period of about 12 weeks. The inclusion into the randomized period will start around March, 2006 and stop around June 1, 2006. The active 12 months intervention period will be finished at around June 1, 2007 and the final 24-month follow up visit will complete the study around June 2008. Subsequent analysis, data handling and writing of publications will be performed in years 2008-2010.

7. INVESTIGATION AND METHODS

7.1 STUDY VISIT SPECIFIC MEASUREMENTS AND REGISTRATIONS

The study consists of a total of 26 visits for adults and 18 for children. These are listed below. Screenings will be conducted by research coordinators following a written protocol. Candidates for participation will be scheduled for an initial interview. In the face-to-face interview with the family, all family members will be informed of the nature and requirements of the study, including randomization to treatment conditions. Each family member must give informed written consent (adults and children where appropriate) before any study-related procedures are carried out.

7.1.1. ADULT VISITS

7.1.1.1 Visit 1, Screening (between week -12 and -9)

Subjects/families will be allocated a screening number before the screening registrations are carried out. The screening registrations will include:

- Medical history including demographic data and physical examination
- Screening and Eating disorders Questionnaires
- Anthropometric measures: height, weight, etc
- BP
- Fasting glucose will be measured using a finger prick test
- Urine stick test

7.1.1.2 Visit 2, CID 1 (between week -12 and -9)

This baseline examination will register the following parameters:

- Anthropometric measures including:
    - Weight
    - Waist/hip and sagittal
    - Body composition (DEXA/bioimpedance)
- BP
- Fat biopsy
- Fasting blood samples
- OGTT & VAS scores
- End of day/compliance questionnaire
- Functional questionnaire
- Attitudinal/psychosocial questionnaire
- Food choice questionnaire
- Physical activity scheme
- 3-day diet record including hedonics/hunger questionnaire
- 24-h urine collection

7.1.1.3 Visit 2a (Subgroup a), CID 1a (between week -12 and -9)

- DLW + urine collection
- BMR
- 7-day diet record
- Pedometer

- Meal test
- VAS scores

7.1.1.4 Visit 3-7 during LCD period, (group meetings >3 families) (week -8, -7, -5, -3 and -1)
Registrations include:

- Weight
- Dietary instruction
- End of day/compliance questionnaire
- Adverse events & concomitant medication

7.1.1.5 Visit 3b (Subgroup b) during LCD period, (week -8, day 3)

- Fat biopsy
- Adverse events & concomitant medication

7.1.1.6 Visit 8, randomisation meeting (>3 families) (week 0)
Registrations include:

- Weight
- Dietary instruction
- End of day/compliance questionnaire
- Adverse events & concomitant medication
- Inclusion/exclusion
- Randomisation

7.1.1.7 Visit 9, CID 2 (individual family meeting) (week 0)
This family meeting (children participation is preferred but not required) following randomisation will register the following parameters:

- Anthropometric measures including:
    - Weight
    - Waist/hip and sagittal
    - Body composition (DEXA/bioimpedance)
- BP
- Fat biopsy
- Fasting blood samples
- OGTT & VAS scores
- End of day/compliance questionnaire
- Functional questionnaire
- Attitudinal/psychosocial questionnaire
- Food choice questionnaire
- Dietary instruction
- Adverse events & concomitant medication

7.1.1.8 Visit 9a (Subgroup a), **CID 2a (week 0)**

- DLW + urine collection
- BMR

- 7-day diet record
- Pedometer
- Meal test & VAS

7.1.1.9 Visit 10, individual family meeting (week 2)
Registrations include:
- Weight
- Dietary instruction
- End of day/compliance questionnaire
- Adverse events & concomitant medication
- 3-day diet record including hedonics/hunger questionnaire

7.1.1.10 Visit 11, individual family meeting (week 4)
Registrations include:
- Weight
- Dietary instruction
- End of day/compliance questionnaire
- Adverse events & concomitant medication
- 24-h urine collection

7.1.1.11 Visit 12 & 13, individual family meeting (week 6)
Registrations include:
- Weight
- Dietary instruction
- End of day/compliance questionnaire
- Adverse events & concomitant medication

7.1.1.12 Visit 14, (group meetings >3 families) (week 10)
Registrations include:
- Weight
- Dietary instruction
- End of day/compliance questionnaire
- Adverse events & concomitant medication

7.1.1.13 Visit 15, (group meetings >3 families) (week 14)
Registrations include:
- Weight
- Dietary instruction
- End of day/compliance questionnaire
- Adverse events & concomitant medication
- 24-h urine collection

7.1.1.14 Visit 16 & 17, (group meetings >3 families) (week 18 & 22)
Registrations include:
- Weight

- Dietary instruction
- End of day/compliance questionnaire
- Adverse events & concomitant medication

7.1.1.15 Visit 18, CID 3, end of intervention period 1 (week 26)
This family meeting will register the following parameters:

- Anthropometric measures including:
  - Weight
  - Waist/hip and sagittal
  - Body composition (DEXA/bioimpedance)
- BP
- Fat biopsy
- Fasting blood samples
- OGTT & VAS scores
- End of day/compliance questionnaire
- Functional questionnaire
- Attitudinal/psychosocial questionnaire
- Food choice questionnaire
- Dietary instruction
- Adverse events & concomitant medication
- 3-day diet record including hedonics/hunger questionnaire
- 24-h urine collection

7.1.1.16 Visit 18a (Subgroup a), **CID 3a (week 26)**

- DLW + urine collection
- BMR
- 7-day diet record
- Pedometer

7.1.1.17 Visit 19-24, family meeting (week 28, 32, 36, 40, 44 & 48)
Registrations include:

- Weight
- Dietary instruction
- End of day/compliance questionnaire
- Adverse events & concomitant medication

7.1.1.18 Visit 25, CID 4, end of intervention period 2 (week 52)
This family meeting will register the following parameters:

- Anthropometric measures including:
  - Weight
  - Waist/hip and sagittal
  - Body composition (DEXA/bioimpedance)
- BP
- Fasting blood samples
- OGTT & VAS scores
- Dietary instruction

- Adverse events & concomitant medication
- 3-day diet record
- 24-h urine collection

7.1.1.19 Visit 26, family follow-up meeting (week 104)
Registrations include:
- Weight
- Waist/hip and sagittal
- Dietary instruction (adherence)
- BP
- Adverse events & concomitant medication

7.1.1.20 Early termination
The registrations include (listed by priority):
- Date & reason for discontinuation
- Adverse events & concomitant medication
- Physical examination including BP (only if early termination is due to adverse event)
- Weight
- Body composition (DEXA-scanning/bioimpedance)
- Blood samples
- End of day/compliance Questionnaire
- RTD4 Questionnaires?
- Waist/hip and sagittal measurement
- Nutritional counselling

There will be a maximum volume of 110 ml blood taken on each CID day and max. 2g adipose tissue.

BIDR questionnaire

7.1.2. VISITS FOR CHILDREN (NB: No questionnaires are completed by children)

7.1.2.1 Visit 1, Screening (between week -12 and -9) (all children)
Subjects/families will be allocated a screening number before the screening registrations are carried out. The screening registrations will include:
- Medical history including demographic data and physical examination
- Anthropometric measures: height, weight, etc

7.1.2.2 Visit 2, CID 1 (between week -12 and -9)
This baseline examination will register the following parameters:
- Anthropometric measures including:
    - Weight (all children)
    - Height (all children)
    - Waist/hip (overweight children only)
    - Body composition (DEXA/bioimpedance) (overweight children aged >12 years only)
- Physical activity scheme

- 3-day diet record (overweight children only)
- Fasting blood samples (all children)
- BP (overweight children only)

7.1.2.3 Visit 3, randomisation family meeting (week 0)
NB: Children participation preferred but optional
Registrations include (all children):

- Weight
- Height
- Dietary instruction
- Adverse events & concomitant medication

7.1.2.4 Visit 4, family meeting (week 2)
Registrations include (all children):

- Weight
- Height
- Dietary instruction
- Adverse events & concomitant medication

7.1.2.5 Visit 4, CID 2 & family meeting (week 4)
This family meeting will register the following parameters:

- Anthropometric measures including:
    - Weight (all children)
    - Height (all children)
    - Waist/hip (overweight children only)
    - Body composition (DEXA/bioimpedance) (overweight children aged >12 years only)
- Fasting blood samples (all children)
- Dietary instruction (all children)
- BP (overweight children only)
- Adverse events & concomitant medication (all children)
- 3-day diet record (overweight children only)

7.1.2.6 Visit 5 & 6, family meeting (week 6 & 10)
Registrations include (all children):

- Weight
- Height
- Dietary instruction
- Adverse events & concomitant medication

7.1.2.7 Visit 7, family meeting (week 14)
Registrations include (all children):

- Weight
- Height
- Dietary instruction
- Adverse events & concomitant medication

7.1.2.8 Visit 8 & 9, family meeting (week 18 & 22)
Registrations include (all children):

- Weight
- Height
- Dietary instruction
- Adverse events & concomitant medication

7.1.2.9 Visit 10, CID 3, end of intervention period 1 (week 26)
This family meeting will register the following parameters:

- Anthropometric measures including:
    - Weight (all children)
    - Height (all children)
    - Waist/hip (overweight children only)
    - Body composition (DEXA/bioimpedance) (overweight children aged >12 years only)
- Fasting blood samples (all children)
- Dietary instruction (all children)
- Adverse events & concomitant medication (all children)
- BP (overweight children only)
- 3-day diet record (overweight children only)

7.1.2.10 Visit 11-16, family meeting (week 28, 32, 36, 40, 44 & 48)
Registrations include (all children):

- Weight
- Height
- Dietary instruction
- Adverse events & concomitant medication

7.1.2.11 Visit 17, CID 4, end of intervention period 2 (week 52)
This family meeting will register the following parameters:

- Anthropometric measures including:
    - Weight (all children)
    - Height (all children)
    - Waist/hip (overweight children only)
    - Body composition (DEXA/bioimpedance) (overweight children aged >12 years only)
- Dietary instruction (adherence) (all children)
- Fasting blood samples (all children)
- Adverse events & concomitant medication (all children)
- BP (overweight children only)
- 3-day diet record (overweight children only)

7.1.2.12 Visit 18, family follow-up meeting (week 104)
Registrations include:

- Weight (all children)
- Height (all children)
- Waist/hip (overweight children only)

- Dietary instruction (adherence) (all children)
- Adverse events & concomitant medication (all children)

7.1.2.13 Early termination (overweight children only)
The registrations include (listed by priority):

- Date and reason for discontinuation (all children)
- Adverse events & concomitant medication
- Physical examination including BP (only if early termination is due to adverse event)
- Weight
- Body composition (DEXA-scanning/bioimpedance)
- Blood samples
- Diet registration (3 days)
- Waist/hip measurement
- Nutritional counselling

There will be a maximum volume of 25 ml blood taken on each CID day from children.

7.2 METHODS OF PRIMARY EFFECT PARAMETERS

7.2.1 BODY WEIGHT

Weight will be recorded to the nearest 0.1 kg. On CID 1-4, the subject must only be wearing underwear and weight should be measured in the morning in a fasting condition and with an empty bladder. At all other visits, the only requirement is that subjects should be weighed without coat & shoes. The weight will be recorded in source data at each visit using the same (or similar) calibrated scale.

7.2.2 HEIGHT

Height will be measured with a wall-mounted stadiometer to the nearest 0.5 cm without shoes. Height will be measured for adults once at screening, and at each visit for children.

7.2.3 BODY COMPOSITION

Body composition will be determined for adults and overweight children 4 times during the study, on CID 1-4, by DEXA or bioimpedance
A DEXA scanner (Lunar Radiation Co., Madison, Wisconsin, USA) will be used to determine lean tissue mass (LTM), fat mass (FM), bone mineral content (BMC) and total body weight (TBW). All scans will be performed using the appropriate scan velocity, depending on body weight and sagittal height. The expected scan time is 5-10 min. The subjects will receive 0.04 – 0.08 mrem of radiation pr. scan.
All subjects will be scanned in a fasting condition and only wearing light clothing. The total scan area will measure 61 cm x 196 cm. For subjects exceeding the grid, DEXA scans will be performed by excluding the outermost part of the arm and/or the thigh.
The subjects will be wrapped in a broad belt, made of coarse tightly woven cloth and closed using Velcro straps to maintain their position during the scan.
The same (or similarly competent) scan-operator will be used throughout the study.

The same scan-analyzer will be used throughout the study, to avoid variation in analysis of the scan results. Quality control and calibration check using the Lunar Aluminum Spine Phantom (LSP) will be performed at least once a week.
DEXA subject information will be handed to the subjects.

7.3 METHODS OF SECONDARY EFFECT PARAMETERS

7.3.1 WAIST AND HIP CIRCUMFERENCE AND SAGITTAL MEASUREMENT

The waist and hip circumference will be determined for adults and overweight children 5 times during the study, on CID 1-4, and during follow-up (week 104). Adults will also have sagittal measurements.
The circumference will be measured to the nearest 0.5 cm with the same tape, if possible, with the subject standing. The subject must only be wearing underwear. The waist measurement will be taken midway between the lower rib and iliac crest at the umbilicus. The waist circumference should be measured in a fasting condition in the morning and with an empty bladder. The subject should breathe normally and the measurement should be taken when the subject is breathing out. The hip circumference will be measured as the largest circumference in the area around the buttocks.

7.3.2 BLOOD LIPID PROFILE

Blood lipids are determined on CID 1-4 for adults only.
Measurements of fasting total HDL, total LDL, VLDL cholesterol, total cholesterol and triglycerides are performed.

7.3.3 BLOOD COAGULATION FACTORS, HORMONES, PEPTIDES & OTHERS

In adults, fasting blood samples for the analysis of coagulation factor VIIc, fibrinogen, C-reactive protein, adiponectin, ghrelin and other relevant peptides, hormones or markers of appetite regulation, body metabolism, inflammatory responses, or obesity related diseases will be taken on CID 1-4. Blood for analysis of various adipokines/cytokines and peptides will be taken on CID 1-3 (adults only), and blood for DNA extraction will be taken on CID 1.

In children, fasting blood samples for the analysis of glucose, insulin, Insulin-like Growth Factor-1 (IGF-1), IGF Binding Protein-3 (IGFBP-3) or other relevant markers for growth, appetite regulation, glucose homeostasis, cardiovascular risk etc. will be taken.

7.3.4 GLUCOSE SENSITIVITY

An OGTT will be performed on adult subjects on CID 1-4. Each subject will take a load of 75 g glucose within a 5 min period. The subjects will remain semi-recumbent and resting throughout the procedure, and must fast (except for 1 dL of water) until completion of the test. Blood samples will be obtained for glucose and insulin at 0, 30, 60, 90 and 120 minutes after drinking the liquid glucose solution. The 0 and 60 min samples will also be analyzed for small and large-sized peptides (i.e. peptidomic/proteonomic analyses).
Information about the OGTT-procedure will be given out to the subject. During the OGTT subjective appetite ratings will be made using Visual Analogue Scales (VAS).

7.3.5 BLOOD PRESSURE

Systolic and diastolic BP will be recorded by an automatic device after 5-10 min while resting in a supine position at screening (visit 1), CID 1-4 and during follow-up (week 104) (adults) and on CID 1-4 (overweight children).

7.3.6 PHYSICAL ACTIVITY

This is a 2. outcome measure therefore need to know – how often & what activity scheme used.

7.4 METHODS OF ADIPOSE TISSUE SAMPLING

An adipose tissue sample will be taken on CID 1-3 and 3 days into the LCD diet (Subgroup b only). This will be analysed for mRNA content (genome wide transcriptomic analyses) by RTD line 2, coordinated by Dominique Langin (INSERM UPS U586, BP 84225, 31432 TOULOUSE Cedex 4, France).

7.5 SUBGROUP MEASURES

7.5.1 METHODS FOR DLW ANALYSIS

On CID 1-3 subjects will be dosed with doubly labelled water to estimate daily energy expenditure over 14 days. Urines will be collected before DLW dosing, at 2 and 4 hours post-dose and one aliquot at 11 am on days 13 and 14.
Subjects will receive a stable isotope dose of approximately 0.1g D_2O (99%) and 1g H_2^{18} (10%) per kg body weight.

7.5.2 METHODS FOR MEASUREMENT OF BMR

On CID 1-3, respiratory measurements of O_2 and CO_2 will be performed by ventilated hood measurements.

7.5.3 PHYSICAL ACTIVITY & 7-D DIET REGISTRATION

On CID 1-3, physical activity in Subgroup a will be assessed by use of pedometers. During each measurement period subjects will undergo a 7 day recorded food intake, during which time they will wear an activity monitor and assess (once at the end of each day) motivation to eat and mood using a small computerised line-scale system.

7.5.4 MEAL TEST

A meal test will be performed with subjects in Subgroup a on CID 1-2. The subjects will remain semi-recumbent and resting throughout the procedure, and must fast (except for 1 dL of water) until completion of the test.
Information about the meal test procedure will be given out to the subjects. During the meal test subjective appetite ratings will be made using Visual Analogue Scales (VAS).

7.6 METHODS OF SAFETY

NB: The study and study procedures are not expected to result in any adverse effects but if so, these adverse effects are anticipated to be minor. As with any research study, there is a possibility of unforeseeable risks that cannot be anticipated by either the investigators or participants. The study may include risks that are unknown at this time. Physical examinations will be performed before (visit 1 at inclusion) and during the study period if suspicion of side effect or other illnesses are presented.

7.6.1 PHYSICAL EXAMINATION

A brief basic physical examination including questions about medical history, previous drug use, etc. will be performed by a Medical Doctor. The examination results obtained will be recorded as normal/abnormal.

7.6.2 URINE SAMPLE (SPOT)
Spot urine samples will be taken from every adult subject on CID 1. The urine will be analyzed (by stix) to assess the amount of protein, glucose etc. present.

7.6.3 SUB-STUDY FOR SAFETY ASPECT OF HIGH-PROTEIN DIET IN CHILDREN AND ADULTS

This specific protocol will assess the potential risks associated with the high protein diet in both children and adults and will examine potential dietary effects on growth and metabolism in children and adolescents. Potential adverse effects on urinary calcium loss, bone loss or renal function will be assessed. The concentrations of serum urea (carbamide), albumin, osteocalcin and creatinine, and urinary albumin, creatinine and U-NTx will be measured in all intervention children on CID 1, 3 and 4. Serum urea and creatinine, and urinary albumin will in addition be measured on CID 2. Serum urea and urinary albumin, creatinine and U-NTx will also be measured in adults on CID 1, 3 and 4. The safety issue will also be addressed at follow-up (week 104) and the general health status of both children and adults will be assessed and certain parameters (e.g. height and weight in children) again measured. Any observed adverse effects (this applies to both children and adults) will be reported to the Ethical Officer in the DiOGenes Project Board (Professor Ruud ter Meulen, Director of the Institute for Bioethics, Department of Caring Sciences of the University of Maastricht, Netherlands) and action will be taken based on the decisions taken by the Project Board.

7.7 METHODS OF COMPLIANCE

7.7.1 NUTRITIONAL COUNSELLING
Nutritional counselling will be given at all main-group visits during and after the LCD diet.

7.7.2 3-DAY DIET REGISTRATION
On CID 1-4, adult subjects and overweight children will be instructed to register their daily diet in a food diary for 3 days. One of the days should be a day at the weekend. The subjects should not let the registration of food influence their daily diets. Instructions for the diet registrations will be the responsibility of a nutritionist.

7.7.3 REGISTRATION OF THE DIET DURING THE INTERVENTION PERIODS
During the dietary intervention period 1, all subjects (except those in the control group) will be supplied with all their food to take home from a purpose built shop at the Department of Human Nutrition.
Each food item will possess a bar code, and food items received by the subject will be registered in a database in the shop. This will allow accurate registration of the dietary intake with regard to total energy, energy from protein, fat and carbohydrates, simple sugars, fibre content, alcohol and energy density (calculated without drinks) etc.
Alcohol will not be available from the shop and the subjects are instructed to report their alcohol intake on a weekly basis (i.e. units (12g)/week). The subjects will be instructed to report any undamaged or uneaten food items in the shop within 14 days of purchase.

7.7.4 BLOOD SAMPLES
On CID 1-4, blood samples will be taken for analysis of specifically chosen markers that are expected or shown to differ in the dietary regimens (adults).

7.7.5 24-H URINE SAMPLES
24-h urinary collection (with PABA control) will be performed from every adult subject on CID 1, visit 11 and 14, CID 3 and 4 to assess urinary nitrogen and other relevant markers such as C-peptide. Subjects will be given written instruction on the 24h urinary sampling procedure.

7.8 ADVERSE EVENTS AND REGISTRATION OF ADVERSE EVENTS
An AE is any unfavourable, unintended event (symptom, disease, or abnormal laboratory finding) reported by a subject or observed by the investigator during the trial, which does not necessarily have a causal relationship with the treatment. Any relevant AE and spontaneously reported symptoms must be recorded in the source data at every visit after the initial visit. Follow-up of any AE reported before the last day of the intervention should be performed according to medical indications. All AE will be recorded in the source data with information about the severity, frequency, date of onset, duration and action taken regarding participation in the study. In addition, the investigator must record the relationship between the AE and the intervention and the subject outcome.
The severity of the AE is recorded according to the following scale: Mild = Awareness of sign or symptom, but easily tolerated, Moderate = Discomfort sufficient to cause interference with normal activities and Severe = Incapacitating, with inability to perform normal activities.
A serious adverse event (SAE) is any untoward medical occurrence that:

- Results in death
- Is life-threatening
- Requires patient hospitalization or prolongation of existing hospitalization
- Results in persistent or significant disability or incapacity
- Is a congenital anomaly / birth defect

The subject must be admitted to hospital in order to be considered as receiving hospital treatment. Hospitalization itself and surgical diagnostic procedures are not AE. Hospitalization for elective surgery in a pre-existing condition that has not worsened during study participation is not an AE.
A distinction should be drawn between serious and severe adverse events. A severe event is a major event of its type, but is not necessarily considered serious. For example, nausea, which persists for several hours, may be considered severe nausea, but not a SAE. On the other hand a stroke, which results in, only a limited degree of disability may be considered a mild stroke, but would be a SAE. Thus, a serious or non-serious event also refers to the nature of the event, in addition to the severity of an event.
In case of a SAE the responsible study investigator (i.e. Arne Astrup, RVAU, Denmark, Coordinating Centre for RTD1) must be informed immediately by telephone and/or in writing, within 24 hours.
At least the following data are required for an immediate/expedited SAE report:

- Patient identification (initials, date of birth, patient number)
- Description of the SAE (symptoms, diagnosis). Every clinical finding in this context has to be reported completely.
- Seriousness of the SAE ("mild", “moderate”, "severe") and reasons for the decision

- Causal relationship with the study participation ("probable", "possible", "not related", "not assessable")

In case of death during the study, a report of a post mortem examination has to be made.
The investigator will notify the Ethics Committee according to the ICH-Guidelines “Note for Guidance on Clinical Safety Data Management: Definitions and Standards for Expedited Reporting” and “Note for Guidance on Good Clinical Practice” (ICH GCP).

7.8.1 REGISTRATION OF CONCOMITANT TREATMENT

Relevant concomitant treatment must be registered in the source data. All concomitant treatments that are necessary for the subject's welfare and which do not fall outside the terms of the protocol may be continued during the trial. Increase in dosage as well as introduction of new medications during the trial must also be reported as an AE.

7.8.2 PREGNANCY

Subjects who become pregnant during the study will be excluded (but the rest of the family will be allowed to continue).

7.9 STUDY MATERIAL & QUESTIONNAIRES (ADULTS ONLY)

7.9.1 LCD DIET

The diet used for the 8-week LCD weight loss period will be purchased from a manufacturer of LCD diets. The items used will include powder diets and/or snack bars.

7.9.2 DIETARY INTERVENTION PERIOD 1

All dietary items will, if possible, be sponsored by local food manufacturers.

7.9.3 SCREENING QUESTIONNAIRE

This includes questions relating to previous dieting experience, health, education, weight and lifestyle habits.

7.9.4 EATING DISORDER QUESTIONNAIRE

This will be used during screening to identify subject eating disorders.

7.9.5 FUNCTIONAL QUESTIONNAIRE

This will be used on CID 1-3 to measure possible changes in mental performance (cognitive functioning) during and after weight loss.

7.9.6 ATTITUDINAL/PSYCHOSOCIAL QUESTIONNAIRE

This will be used on CID 1-3. It includes questions relating to behavioural and psychosocial factors (self efficacy, family coherence, motivation, etc), behaviour related to weight control, a Quality of Life questionnaire and the Stunkard 3-factor eating questionnaire (restraint, disinhibition and hunger).

7.9.7 FOOD CHOICE QUESTIONNAIRE

This will be used on CID 1-3 to measure changes in food choices or preferences relating to the degree of satiety, whether sweet or savoury etc. It will be administered to 65 subjects at each centre during the OGTT (before and 30 min after the glucose load) and to 30 subjects (subgroup a) during

the meal test (before and 30 min after the meal). The questionnaire has 2 components: a Forced Choice Photographic Questionnaire and a Food Preference Checklist.

7.9.8 HEDONICS/HUNGER QUESTIONNAIRE
This scheme is part of the food diaries and will ask questions about social factors related to eating.

7.9.9 END OF DAY QUESTIONNAIRE
This will be used on CID 1-3 and weighing days approximately every other week. It will be completed in the subject's own home (on their own computer) at the end of the day and will ask questions relating to hunger, motivation, mood and subjective sensations/experiences.

7.9.10 BALANCED INVENTORY OF DESIRABLE RESPONDING (BIDR) QUESTIONNAIRE
It measures an individual's tendency to give socially desirable responses.

7.9.11 VAS SCHEMES
Subjective appetite ratings (hunger, satiety, fullness, prospective food consumption etc) are measured using computerised VAS scores (100mm scale). The scales will be completed on CID 1-3 during the OGTT and meal test (Subgroup a) at specific time points.

8. DATA MANAGEMENT

8.1 DATA QUALITY ASSURANCE
All study data will be entered as source documents. Data recorded in these documents will be collected in accordance with good clinical practice (GCP). All data management will be carried out in accordance with the protocol, GCP and ICH guidelines.
A central Data Hub (RTD6, coordinated by Dr Claus Holst, Copenhagen University Hospitals, Danish Epidemiology Science Centre, Copenhagen, Denmark) will facilitate the integration between RTD lines, by standardization of protocols and data collation, storage of (and access to) RTD line data, rapid and detailed analysis of complex data sets.

8.2 STATISTICAL PROCEDURES
All statistical analysis described in this protocol will be performed by the investigator. All analysis and tabulations will be performed using the latest release of the SAS® or SPSS software or similar statistical software. Appropriate descriptive statistics will be presented for each variable; n=number of patients, mean, median, SD and min-max for continuously distributed variables, and rates and percentages for categorical variables. Statistical tests will be performed using 5% as the nominal level of significance and interval estimates will be constructed using 95% as the level of confidence. Statistical and analytical methods, and sample size considerations are discussed in the following sections. The investigator will make the decisions regarding subjects, or individual values belonging to a patient, to be excluded from the analysis. Subjects will only be excluded from the efficacy analysis when the uses of these are considered a protocol violation, putting the scientific aspects of the trial at risk. The variables that will be statistically tested are given in the Sections concerning primary and secondary variables.

8.2.1 OBJECTIVES OF ANALYSES AND STUDY VARIABLES

To estimate the long term effects (week 26, week 52 and week 104) of an *ad libitum* diet with high or normal protein content and with high or low GI, compared to a control diet.

8.2.2 MAIN OUTCOME MEASURES

The main outcome measures in this study for adults are body weight loss maintained (kg), changes in body composition (DEXA/bioimpedance), proportion of subjects maintaining > 0, 5 and 10% of initial weight loss, and drop-out rate. Main outcome measures for children are changes in the proportion of overweight and obese children at 6 and 12 mo, and changes in age-associated BMI, % whole body fat and drop-out rate.

8.2.3 SECONDARY OUTCOMES

Secondary outcomes (adults) are reduction in abdominal obesity, changes in risk factors of type-2 diabetes and CVD, appetite/satiety hormones, waist-hip and sagittal measurements and physical activity. Secondary outcomes for overweight children are reduction in abdominal obesity, changes in blood parameters and waist-hip circumference ratio, and changes in physical activity.

Other secondary aims of the study (in collaboration with other RTD lines) are to identify biological, genetic and psychological traits that determine an individual's (or family's) response to the diet interventions. Biological traits (including age, gender, body weight and composition, family size, and genetic make-up) will be evaluated as potential moderators of response. Psychological traits (including satiety, diet satisfaction, food craving, dietary restraint, disinhibition, and perceived hunger and quality of life) will be evaluated as potential moderators and/or mediators of response. Interaction terms between diet and biological/psychological traits will be used to examine potential moderators, and tests of direct and indirect effects will be used to test whether psychological traits mediate the relationship between diet and weight regain (e.g., What proportion of the effect of diet on weight regain can be explained by the diet's effect on satiety?). Mediation models will only be considered if a significant effect of dietary intervention is observed in the primary analysis.

8.2.4 STATISTICAL METHODS FOR THE PRIMARY OBJECTIVES

Analysis of Covariance (ANACOVA) will be used to compare the main outcome measures in the intervention groups at week 26 and week 52 using the data from CID2 (after weight-loss, adults) or CID1 (children) as covariate.

<u>Handling of missing values</u>

Since the sample size estimations take into account the drop-outs during the study, sufficient data should be obtained.

The primary analysis of data will be an "intention to treat" analysis (with only actual values presented) and will include only subjects having completed the study. We will also perform sensitivity analyses by examining different models of missing data and describe the range of possible outcomes based on the various models. For example, we will perform a "baseline carried forward" analysis and a "last observation carried forward" analysis as part of the sensitivity analysis.

8.2.5 PRIMARY HYPOTHESIS

The following primary hypothesis can be applied:

H_0: The 1° outcome (weight, etc) is equal in the intervention groups at week 26 and week 52, when controlling for the value in CID2 (adults) or CID1 (children).

vs.

H_1: The 1° outcome is different in the intervention groups at week 26 and week 52, when controlling for the value in CID2 (adults) or CID1 (children).

The changes in continuously distributed variables from baseline to week 26 and week 52 within treatment groups will be tested with the paired t-test or the Wilcoxon-Signed-rank test, depending on distribution (all tests two-sided).
All categorical variables will be expressed in contingency tables and analyzed using Fisher's exact test. Survival analysis using the Kaplan-Meier plot and the log-rank test will be used to investigate time to subject withdrawal.
All null hypotheses comparing groups assume equality between the treatment groups while the alternative hypotheses assume that there is a difference (two-sided). All within-group null hypotheses assume no change from baseline to week 26 or week 52 while the alternative hypotheses assume that there is a change (two-sided).

8.2.6 STATISTICAL METHODS FOR SECONDARY OBJECTIVES

ANCOVA will be used to compare the secondary variables at week 26 between treatment groups using the relevant data from CID2 (after weight-loss, adults) or CID1 (children) as covariate.

8.2.7 SAMPLE SIZE ESTIMATION

The primary aim of the study is to investigate the long-term efficacy in terms of change in body weight from baseline to study end. The sample size for the proposed study was chosen to provide sufficient power to detect differences in 26-week weight regain (for primary participants) due to the effects of the different diets. The sample estimation is based on the difference between the intervention and control groups regarding weight change (kg) from baseline to week 26. In the null hypothesis we assume that the interventions groups 1 - 4 are of equal weight at week 26 compared to baseline, while the alternative hypothesis assumes that there exists a difference in weight between the treatment groups. By this covariate method we are able to test the change from baseline to week 26 while controlling for between-group differences in the baseline value.
It is anticipated that weight differences will be smallest between the low vs. high GI diets compared to the low protein vs. high protein diets. Hence the sample size has been calculated on the basis of a previous intervention study on GI (18), which found a non-significant difference in body weight change of 0.6 kg between groups using the unpaired t-test. We assume a 0.4 kg higher weight loss based on 6-month treatment, compared to the 10-week previous intervention study (18). Thus we consider that a weight loss difference of 1 kg is a clinically relevant weight loss and a priori we assume a SD of 2.01 kg.
When assuming a difference between control and intervention group of 1.0 kg (SD 2.5 kg), at least 80% statistical power and a significance level of 5%, N=125 participants per group are required (unpaired t-test). In this model, any effect size above 0.72 kg gives a power of more than 80%.

As most other studied variables in the study are secondary objectives and because the outcome of most of these variables are not easily comparable to previous studies due to the novel design of the study, there are no calculations on sample size for variables other than the primary efficacy parameter, which is weight change to week 26.
Thus, in terms of final outcome, it is accepted that post-hoc analysis will identify that not all measures of other primary or secondary outcomes will necessarily have had the appropriate sample size.

9. ETHICAL ASPECTS

9.1 ETHICS COMMITTEE REVIEW

Prior to starting the study, the investigator will submit the study protocol with the supporting documentation to the local/regional Ethics Committee. The trial is not allowed to start until the local/regional Ethics Committee has approved the protocol, the constitution of which satisfies the local regulatory guidelines. Approval must be obtained in writing and a copy of this approval will be kept at the trial sites.

The Ethics Committee must be consulted whenever new events require a significant change in the protocol or its appendices during the study.

9.2 ETHICAL CONDUCT OF THE STUDY

The study will be conducted in agreement with the relevant international conventions and declarations, including latest versions of:

- The Declaration of Helsinki.
- The Danish Central Scientific Ethical Committee, Guidelines on biomedical experiments,
- UN Convention on the Rights of the Child

The subjects will be insured via the public patient insurance.

9.3 POTENTIAL BENEFITS OF THE PROPOSED STUDY & SAFETY ASPECTS

The benefits to the subjects studied in this research protocol, and to society at large, far surpass the risks. The Diogenes project will have great potential impact on future research in the development of obesity in general, and in the relationships between diet and the development of obesity in general. This study is not designed to treat an illness however subjects in the proposed research will receive several immediate benefits in addition to weight loss. The most important of these is repeated assessment and monitoring of several health factors including BMI, blood lipids, blood pressure, and body composition. The results of these assessments will be made available to the participants. Obesity is an epidemic within Europe and the information obtained from this research might help with future public health strategies and policies for the prevention of weight gain. If successful, this intervention could support the concept that higher levels of dietary protein and low GI foods can play a role in prevention of weight gain and in promotion of weight loss maintenance.

The discomfort of subjects enrolled in the intervention study is in all cases anticipated to be mild and temporary. Risks involved in participation are limited to slight discomfort related to blood sampling/injection, and in cases involving sampling of adipose tissue biopsies (adults only) may involve minor haematomas at the site of sampling. Considering the potential treatment benefits involved in securing genetic information that may lead to the discovery of obesity-relevant genes, we believe that these investigations are justified. The potential risk associated with the dietary components of the intervention study with respect to CVD and diabetes type 2 in adults will be fully addressed during the programme.

9.3 PARTICIPATION OF CHILDREN

The inclusion of children in the dietary intervention is expected to produce a health benefit for the overweight members of this group in terms of weight loss or reduced weight gain. It is considered important to include the children in the intervention and investigate the diet within the whole family as some studies have demonstrated that the family based approach to body weight management is

more effective at helping overweight children to lose weight. Measurements for the children will be minimised, however it will necessary to take some blood samples in order, in particular, to fully address the safety aspect of the diet. This will need to be performed in a sensitive manner to assure cooperation by the children and their parents. Although it is not expected that the high protein diet will pose any risk to either children or adults, nevertheless the effects of such a diet will be carefully monitored and action will be taken in the case of clinically significant AE (see Section 9.8 above). It is important to underline that the high protein diet does not provide more than 28% energy from protein, which is within the acceptable range for both children and adults (15). The Institute of Medicine have also concluded that no clear evidence supports that a high-protein diet increases the risk of CVD or damage to kidneys or bone (15). No adipose tissue biopsies will be taken from children.

Any observed AE during the study (this applies to both children and adults) will be reported to the Ethical Officer in the Project Board and action will be taken based on the decisions taken by the Project Board.

9.4 SUBJECT INFORMATION AND CONSENT

Informed consent must be sought and given freely by each adult subject after being fully informed both verbally and in writing of the course and the aims of the study, effects and possible side effects and risks of study participation. Separate consent forms will be supplied for the sub-studies (subgroup a and b), accompanied by the appropriate explanatory literature. The subject involved must have legal capacity to give consent, must be able to exercise free power of choice and must be provided with a fair explanation of all material information concerning the study.
The subject will be given information on the possibilities of compensation and treatment in case of injury or incapacity attributable to his/her participation in the study. She/he will be assured that the consent may be cancelled and that it is possible to withdraw from the study at any time.
The subject should be told that the participation in the study is confidential, but the subject should be made aware that this participation might be disclosed to a Regulatory Authority.
Both the subject and the investigator will sign the consent form. The subject information consent form should be provided for the subject, a copy being included as an appendix to this protocol.
Informed consent for children under 18 y must be procured from the person(s) having custody. If both parents have custody, consent must be obtained from both of them. Consent must also be obtained from the child itself, if this is not excluded by circumstances. An assessment will be made as to whether the child is mature enough to understand what the experiment involves and what consent means. Even so, the experiment may not be conducted if the child objects to it. If a child is unwilling to undergo (one of) the investigative procedure(s), the investigation will be cancelled, despite initial approval by the parents. The parents will be informed about this in advance and have to agree with this behavioural code. Parents can withdraw their approval of the study of their child(ren) at any moment. A trial subject who is unable to give informed consent is to be informed and be included in a discussion of the experiment to the extent that the subject understands the experimental situation, unless this may harm the subject concerned. In the main, children from the age of 12 and up will be able to meet these requirements. However, a specific assessment must always be made.

9.5 SUBJECT RIGHTS TO TRIAL RESULTS
On the informed consent form, subjects will be able to specify their choice to receive information about genetic markers or findings which may indicate potential or actual health risk. Subjects will be offered counselling if required.

9.6 SUBJECT PRIVACY
The investigator affirms and upholds the principle of the subject's right to protection against invasion of privacy. All source documents and other study related documents will be treated as confidential and will be identified by treatment number, case record form number, subject initials and date of birth. The investigator will hold any information seen as confidential and will not reveal it, except when required by law.

10. SCIENTIFIC PUBLICATION
Scientific publication is primarily the responsibility of the DiOGenes Publications Working Group. Authorship will be included according to the Vancouver Declaration.
Reports will be published in relevant international and national peer-reviewed scientific journals, non-scientific journals or other relevant media.

11. MEDICAL ASSISTANCE

Prof. Arne Astrup MD
Prof. Søren Toubro MD

12. SUBJECT REIMBURSEMENT
No remuneration will be paid to trial subjects for their participation in the dietary intervention. However, reimbursement of travel expenses in certain circumstances may be considered.

13. REFERENCES

1. Saris WHM and Harper A. Diogenes: a multi-disciplinary offensive focussed on the obesity epidemic. Obes Rev 6, 175-176. 2005.

2. Cole TJ, Bellizzi MC, Flegal KM, and Dietz WH. Establishing a standard definition for child overweight and obesity wordwide: international survey. BMJ 320, 1-6. 2000.

3. Astrup A, Grunwald GK, Melanson EL, Saris WHM, Hill JO. The role of low-fat diets in body weight control: a meta-analysis of ad libitum intervention studies. Int J Obes 2000;24:1545-52.

4. Knowler WC, Barrett-Connor E, Fowler SE, Hamman RF, Lachin JM, Walker EA, and et al. Reduction in the incidence of type 2 diabetes with lifestyle intervention or metformin. N

Engl J Med 346, 393-403. 2002.

5. Pawlak DB, Ebbeling CB, and Ludwig DS. Should obese patients be counselled to follow a low-glycaemic index diet? Yes. Obes Rev 3, 235-243. 2002.

6. Willett WC. Eat, drink and be healthy: the Harvard Medical School Guide to healthy eating. 2002. Simon and Schuster.

7. Raben A. Should obese patients be counselled to follow a low glycaemic index diet? No. Obes Rev 3, 245-256. 2002.

8. Pi-Sunyer FX. Glycaemic index and disease. Am J Clin Nutr 76, 290S-298S. 2002.

9. Porrini M, Santangelo A, Crovetti R, Riso P, Tesolin G, and Blundell JE. Weight, protein, fat and timing of preloads affect food intake. Physiol Behav 62, 563-570. 1997.

10. Rolls BJ, Hetherington M, and Burley VJ. The specificity of satiety: the influence of foods of different macronutrient content on the development of satiety. Physiol Behav 43, 145-153. 1988.

11. Mikkelsen PB, Toubro S, and Astrup A. The effect of fat-reduced diets on 24-h energy expenditure: comparisons between animal protein, vegetable protein and carbohydrate. Am J Clin Nutr 72, 1135-1141. 2000.

12. Skov AR, Toubro S, Ronn B, Holm L, and Astrup A. Randomised trial on protein vs. carbohydrate in ad libitum fat reduced diet for the treatment of obesity. Int J Obes 23, 528-536. 1999.

13. Eisenstein J, Roberts SB, Dallal G, and Saltzman E. High-protein weight-loss diets: are they safe and do they work? A review of the experimental and epidemiologic data. Nutr Rev 60, 189-200. 2002.

14. Astrup A, Larsen TM, and Harper A. Atkins and other low-carbohydrate diets: hoax or an effective tool for weight loss? Lancet 364, 897-899. 2004.

15. Institute of Medicine, Food and Nutrition Board. Dietary reference intakes for energy, carbohydrate, fiber, fat, fatty acids, cholesterol, protein and amino acids (macronutrients). 2002. Washington DC, National Academy Press.

16. Due A, Toubro S, Skov AR, and Astrup A. Effect of normal-fat diets, either medium or high in protein, on body weight in overweight subjects: a randomised 1-year trial. Int J Obes 28, 1283-1290. 2004.

17. Foster-Powell K, Holt SH, and Brand-Miller JC. International table of glycemic index and glycemic load values: 2002. Am J Clin Nutr 76, 5-56. 2002.

18. Sloth B, Krog-Mikkelsen I, Flint A, Tetens I, Bjorck I, Vinoy S, Elmstahl H, Astrup A, Lang V, and Raben A. No difference in body weight decrease between a low-glycemic-index and a high-glycemic-index diet but reduced LDL cholesterol after 10-wk ad libitum intake of the low-glycemic-index diet. Am J Clin Nutr 80, 337-347. 2004.
